# The *SpTransformer* Gene Family (Formerly *Sp185/333*) in the Purple Sea Urchin and the Functional Diversity of the Anti-Pathogen rSpTransformer-E1 Protein

**DOI:** 10.3389/fimmu.2017.00725

**Published:** 2017-06-30

**Authors:** L. Courtney Smith, Cheng Man Lun

**Affiliations:** ^1^Department of Biological Sciences, George Washington University, Washington, DC, United States

**Keywords:** *Sp185/333*, multitasking, anti-pathogen, purple sea urchin, *Strongylocentrotus*, echinoderm, invertebrate, intrinsically disordered proteins

## Abstract

The complex innate immune system of sea urchins is underpinned by several multigene families including the *SpTransformer* family (*SpTrf*; formerly *Sp185/333*) with estimates of ~50 members, although the family size is likely variable among individuals of *Strongylocentrotus purpuratus*. The genes are small with similar structure, are tightly clustered, and have several types of repeats in the second of two exons and that surround each gene. The density of repeats suggests that the genes are positioned within regions of genomic instability, which may be required to drive sequence diversification. The second exon encodes the mature protein and is composed of blocks of sequence called elements that are present in mosaics of defined element patterns and are the major source of sequence diversity. The *SpTrf* genes respond swiftly to immune challenge, but only a single gene is expressed per phagocyte. Many of the mRNAs appear to be edited and encode proteins with altered and/or missense sequence that are often truncated, of which some may be functional. The standard SpTrf protein structure is an N-terminal glycine-rich region, a central RGD motif, a histidine-rich region, and a C-terminal region. Function is predicted from a recombinant protein, rSpTransformer-E1 (rSpTrf-E1), which binds to *Vibrio* and *Saccharomyces*, but not to *Bacillus*, and binds tightly to lipopolysaccharide, β-1,3-glucan, and flagellin, but not to peptidoglycan. rSpTrf-E1 is intrinsically disordered but transforms to α helical structure in the presence of binding targets including lipopolysaccharide, which may underpin the characteristics of binding to multiple targets. SpTrf proteins associate with coelomocyte membranes, and rSpTrf-E1 binds specifically to phosphatidic acid (PA). When rSpTrf-E1 is bound to PA in liposome membranes, it induces morphological changes in liposomes that correlate with PA clustering and leakage of luminal contents, and it extracts or removes PA from the bilayer. The multitasking activities of rSpTrf-E1 infer multiple and perhaps overlapping activities for the hundreds of native SpTrf proteins that are produced by individual sea urchins. This likely generates a flexible and highly protective immune system for the sea urchin in its marine habitat that it shares with broad arrays of microbes that may be pathogens and opportunists.

## Introduction

Immune activities in animals that survive the arrays of pathogens with which they share their habitats, display a wide range of innate functions irrespective of whether they also deploy adaptive immunity. The underlying attributes of many genes that act in pathogen detection or anti-pathogen responses typically show significant sequence diversity in the encoded proteins that can be derived from gene diversification mechanisms, mRNA processing that may include posttranscriptional changes, and posttranslational modifications to the proteins. Single copy genes that function in immunity can also display significant sequence diversity through large numbers of alleles in a population. Some examples are genes linked in the fusion/histocompatibility locus in the compound tunicate *Botryllus schlosseri*, and genes in the major histocompatibility locus in mammals and other vertebrates [reviewed in Ref. (1)]. However, many of the genes that encode innate immune functions are expanded into families such as Toll-like receptors and NOD-like receptors in most animals, fibrinogen-related proteins in mollusks, and killer immunoglobulin-like receptors in mammals. Common attributes of immune gene family members include clustering, shared sequences, repeats, plus elevated levels of duplications, deletions, and recombination (2). These attributes typically generate pseudogenes, but also generate new genes that can be expressed and are then subject to selection based on increased host fitness in responses to and protection from pathogens. A gene family with these attributes in the purple sea urchin, *Strongylocentrotus purpuratus*, is the *Sp185/333* gene family, which will be the focus of this review. A recombinant (r)Sp185/333 protein shows multitasking activities with characteristics for binding to different types of microbes and multiple pathogen-associated molecular patterns (PAMPs) (3), and transforms from intrinsic disorder to α helical structure upon binding a target (4, 5). These attributes underlie the new name for this particular recombinant protein from rSp0032, which was based on the cDNA nomenclature as reported by Terwilliger et al. (6), to rSpTransformer-E1 (rSpTrf-E1) that is based on a combination of its structural conformational changes and its E1 element pattern (4). In keeping with maintaining continuity between names for genes and their encoded proteins, the gene family has also been renamed from *Sp185/333* to *SpTransformer* (*SpTrf*) and the general name for the proteins have been changed from Sp185/333 proteins to SpTrf proteins. These updated names will be used in this review and in all future reports on this system.

## Discovery; *SpTrf* Gene Expression and Sequence Diversity of the mRNAs

**Figure 1:**
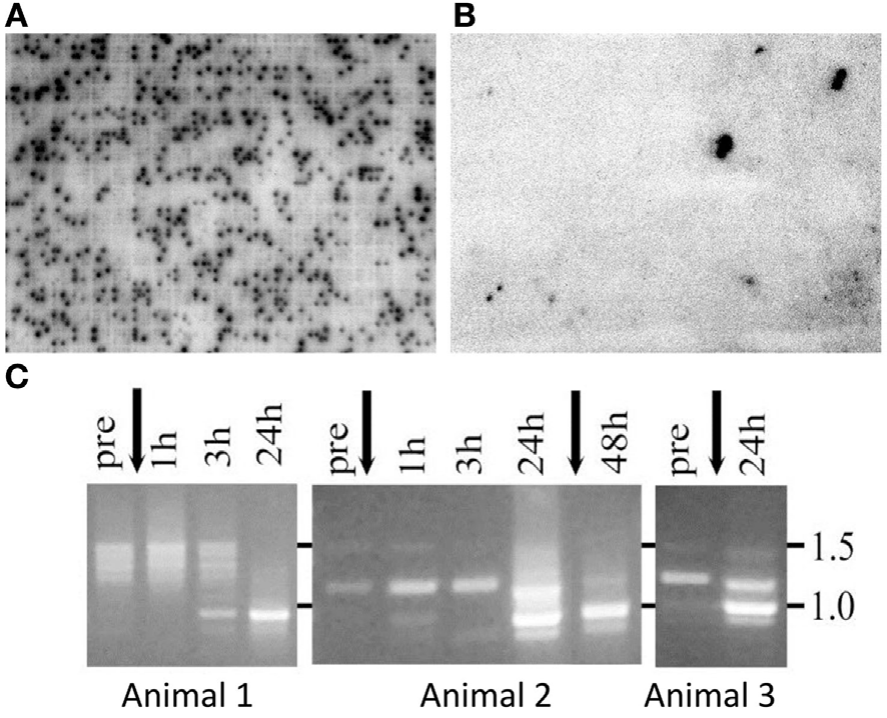
The *SpTransformer* (*SpTrf)* genes are expressed in response to immune challenge. Two arrayed cDNA libraries constructed from coelomocytes: **(A)** collected from six sea urchins after immune challenge by injection of marine bacteria or **(B)** collected from six sea urchins that were not challenged. Individual colonies harboring cDNAs were arrayed into 91,920 separate wells in 240 plates of 384 wells/plate. cDNA inserts for each colony were amplified, spotted onto a nylon filter [for details, see Ref. (10)], and both libraries were screened with a ^32^P-RNA probe constructed from a set of *SpTrf* cDNA clones (9). The activated library has ~5,925 *SpTrf-*positive spots or 6.45% of the library, whereas the non-activated coelomocyte library has 79 *SpTrf-*positive spots or 0.086% of the library. Positive clones are indicated by two spots within a 4 × 4 set of amplified insert cDNA from each clone in the library. **(C)** Coelomocytes collected over time from three immunoquiescent sea urchins and analyzed by RT-PCR show changes in the *SpTrf* amplicon sizes before vs. after one or two injections of lipopolysaccharide (arrows). The major element pattern identified after cDNA insert sequencing is *E2* has an amplicon size of about 935 nt, which is similar to the single band observed at 24–48 h post challenge. Panel **(C)** is reprinted from Ref. (11).

The first reports of *SpTrf* sequences included an expressed sequence tag (EST; equivalent of an RNA-Seq read) from a cDNA library constructed from coelomocytes after challenge with lipopolysaccharide (LPS) (7) and a full-length coelomocyte cDNA sequence identified after challenge with marine bacteria and injury based on results from analysis by differential display (8). Both were noteworthy because of significant upregulated gene expression in coelomocytes in response to immune challenge. When an arrayed cDNA library constructed from immune activated coelomocytes was screened with a subtracted probe specific for mRNAs in LPS-activated coelomocytes, clones identified in the library indicated a striking upregulation in gene expression of these same sequences, and which constituted ~60% of the sequenced clones (9). The names of the original EST and differential display clones, 333 and 185, were used in the original name of the gene family and collection of cDNAs because the deduced protein sequences did not match to any proteins in any other organism and offered no prediction for function. Upon re-screening the arrayed cDNA libraries for clones with *SpTrf* sequences, positive clones constituted 6.45% of the library constructed from bacteria challenged coelomocytes and 0.086% of the non-activated library (Figures 1A,B). This 75-fold increase in gene expression in response to challenge correlates with results from the original Northern blots (8). Comparisons among the cDNA sequences show significant and intriguing sequence diversity that, in addition to the gene expression characteristics, was the basis for additional investigations.

Sea urchins in their normal marine habitat are in constant contact with microbes in the water, on the substrate, and associated with their diet, and healthy animals maintain a constant level of immune activity. However, this immune activity complicates experimental evaluation of immune responsiveness of sea urchins to a particular PAMP or microbe. This problem was resolved by the discovery that when sea urchins are kept in closed, recirculating marine aquaria for more than 6- to 8-months and away from the input of “wild” sea water, they turn down their immune responsiveness and, therefore, have been called immunoquiescent (IQ) (12). Examples of downregulated gene expression in IQ animals include the complement homolog, *SpC3* (12–14), and the *Sp056* gene that encodes the small C-type lectin, SpEchinoidin (11). Consequently, when IQ sea urchins are immune challenged to determine activators of the *SpTrf* genes, expression is induced with one or two injections of LPS (Figure 1C), β-1,3-glucan (a fungal PAMP), double stranded (ds)RNA (polyGC to represent a viral challenge), or injury that includes injection of buffer (11). Prior to challenge or injury in IQ sea urchins, *SpTrf* amplicons are either absent or show a spread of weak bands of about 1.2–1.5 kB (Figure 1C). After challenge, an increase in the intensity of the amplicons is noted and the amplicon sizes change differently among individual animals but tend to focus on a single major size of ~0.9 kB. This indicates a change from diverse or no expression in non-challenged IQ sea urchins to a focus on a major band that likely corresponds with cDNAs of similar size that are the most common version of the *SpTrf* cDNA sequences (see below).

**Figure 2:**
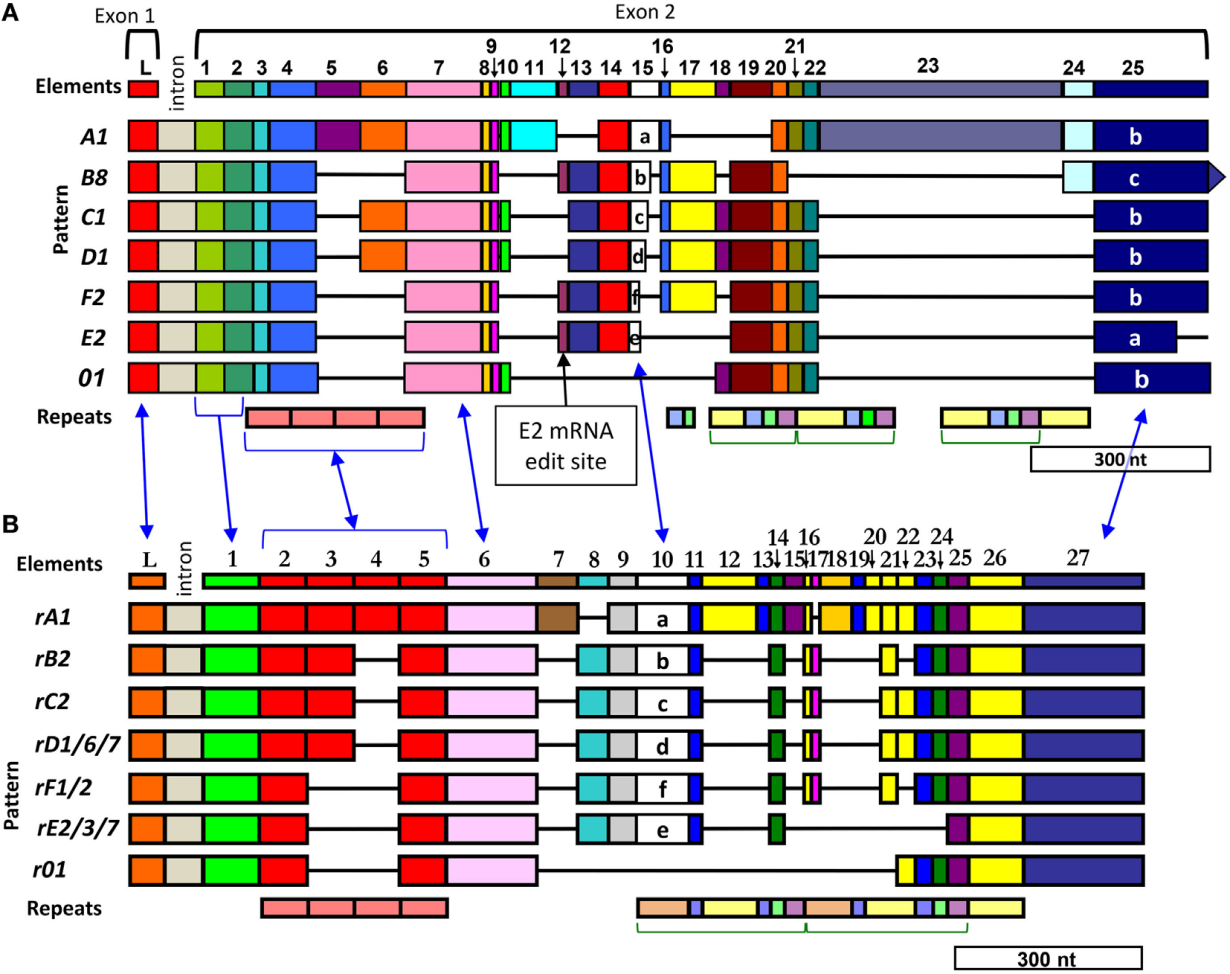
Diversity of elements and repeats in the *SpTransformer* (*SpTrf)* sequences enable two equally optimal alignments. Optimal alignments of the *SpTrf* sequences require the insertion of artificial gaps (black horizontal lines) that delineate individual elements shown as colored blocks [numbered across the top (L, leader)]. The intron is not to scale. Different element patterns are based on the variable presence or absence of elements and are listed to the left of each alignment. The combination of elements defines the element pattern that is named according to the distinctive and highly diverse sequence of element 15 (6). **(A)** The initial alignment employed fragments of cDNA sequences, expressed sequence tags, and full-length cDNA sequences according to Terwilliger et al. (6, 11) and is called a “cDNA-based” alignment. Element 25 in panel **(A)** is listed as three types, a, b, and c, which are defined by the location of the first of three possible stop codons in the element. These three stops are also present in element 27 in panel **(B)** but are not indicated. A very common mRNA editing site in element 12 is indicated for the *E2* element pattern (indicated) that changes a codon to a stop to encode a truncated *E2.1* sequence that omits the histidine-rich region of the protein (see also Figure 4B). **(B)** A subsequent alignment optimizes the edges of the elements with the edges of the repeats according to Buckley and Smith (15) and is called the “repeat-based” alignment. Both types of alignments are feasible with cDNA and gene sequences. The blue arrows between the two alignments indicate identical regions. Repeats are shown at the bottom of each alignment, occur as tandem repeats or interspersed tandem repeats, and are denoted by different colors (type 1, red; type 2, blue; type 3, green; type 4, yellow; type 5, purple; type 6, peach). This figure is modified from Ref. (16).

Automated alignments of the *SpTrf* cDNA sequences fail when using standard alignment programs with default parameters, which forced alignments to be done manually. Challenges for generating alignments are due to the unusual characteristic of the *SpTrf* sequences in which insertions of large artificial gaps are required for optimal alignments. These gaps identify and define recognizable blocks of sequence called *elements* (Figure 2A) (6, 11). The initial alignments were based on the cDNA sequences and identified a maximum of 25 elements, of which, subsets of elements are present as mosaics in individual sequences; no sequences have the full complement of possible elements. Different mosaics of elements are repeatedly identified and are termed *element patterns* and correlate with the sequence variants of element 15. This highly diverse element is present in a range of sizes and is employed as the basis for naming the element patterns of *A* through *G* (Figure 2A). Some sequences do not include element 15 and are termed *0* patterns. Other attributes of the cDNA sequences include repeats identified as tandem type 1 repeats, interspersed repeats of types 2–5, and one to three possible stop codons in element 25 defined as element 25a, b, or c. The swift upregulation of the *SpTrf* genes in response to immune challenge and the striking sequence diversity of the cDNAs strongly suggest that this family has important activities in the sea urchin immune response.

Ongoing and repeated searches of sequence repositories have only identified *Trf* sequences in other euechinoids. In phylogenetic analyses of the euechinoid order within the echinoid class of echinoderms, it clusters separately from the cidaroid order, which is more ancient [for details on echinoderm phylogeny, see Ref. (17, 18)]. Searches of the genome sequences from the euechinoid sea urchins, *Mesocentrotus franciscanus, Strongylocentrotus fragilis* [see (19) for genus revisions in the strongylocentrotid sea urchins], and *Lytechinus variagatus* identify matches to *Trf* genes. A single cDNA sequence has been reported for *Strongylocentrotus intermedius* (20), and 39 *HeTrf* (formerly *He185/333*) gene sequences have been characterized from *Heliocidaris erythrogramma*, another sea urchin species (21). However, searches of the genome sequence of the pencil sea urchin, *Eucidaris tribuloides*, in addition to other cidaroid species and other classes of echinoderms show no matches to *Trf* genes. Given the outcomes of these searches, the *Trf* gene family appears to be a derived character of innate immunity that is present only within the regular euechinoid sea urchins.

## The *SpTrf* Genes are Small, Arranged in Tight Clusters, and have Shared but Diverse Sequences

**Figure 3:**
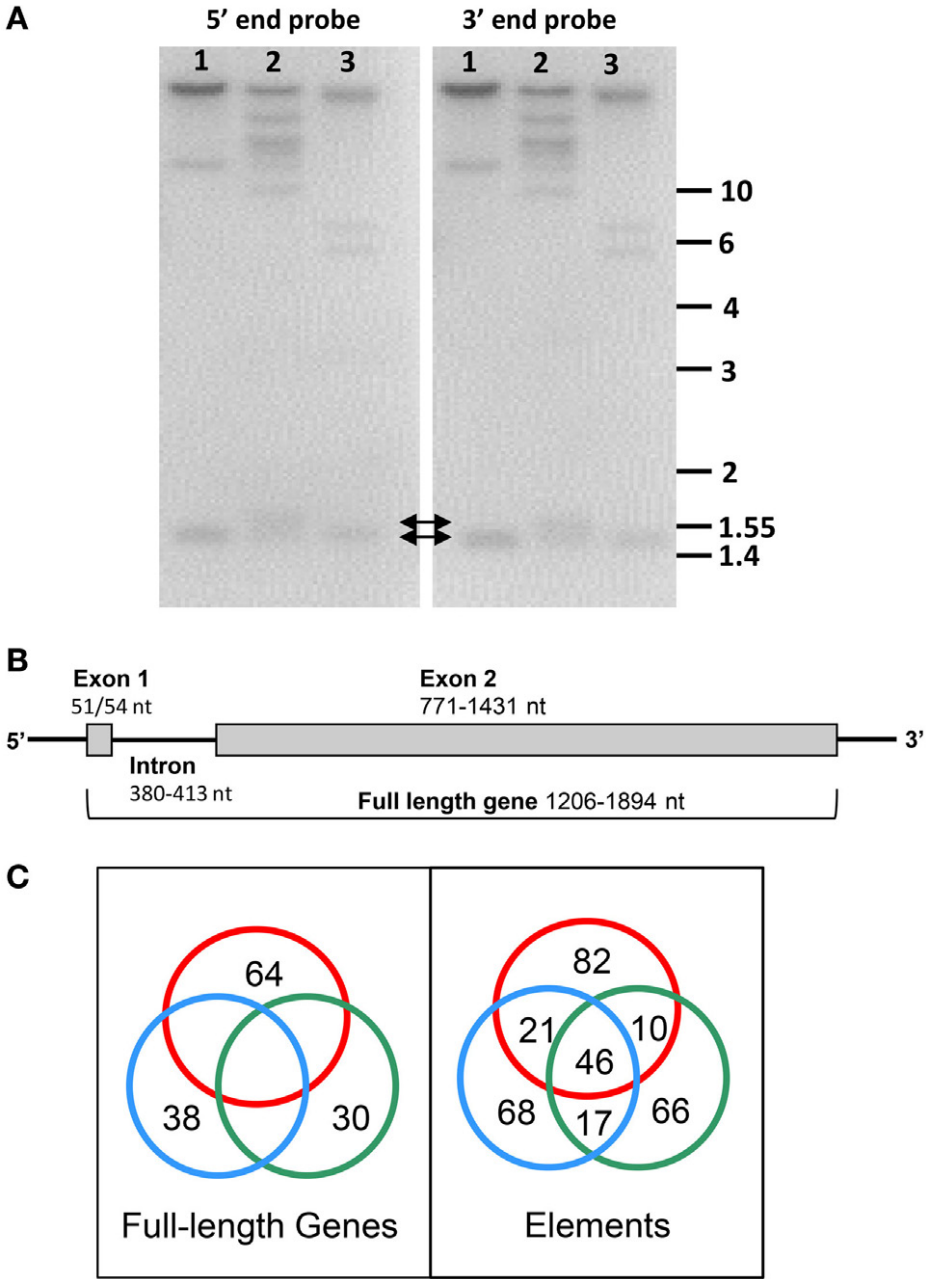
The *SpTransformer* (*SpTrf)* gene family is diverse, but the gene structure is simple. **(A)** Digests of genomic DNA from three sea urchins (1–3) with *Pst*I are shown as duplicate Southern blots that are evaluated with ^32^P-labeled riboprobes spanning elements 1–7 (5′ end) and from elements 7–25 (3′ end) (see Figure 2 for elements). Both probes hybridize to bands of less than 2 kB (arrows) (see Terwilliger et al. (6) for methods). This figure is reprinted from Ref. (16). **(B)** The *SpTrf* genes are small with two exons. Although the genes show significant sequence diversity, their overall structure is generally the same with two exons. This figure is modified from Ref. (2). **(C)** Amplified, cloned, and sequenced genes (171 total) from three sea urchins are represented as red, blue, and green circles in this Venn diagram. Comparisons among nucleotide sequences of the full-length genes within and among sea urchins identified no identical matches (left). However, shared element sequences are present in genes within and among sea urchins (right). Shared sequences are indicated by intersections of the circles. This figure is reprinted from Ref. (16).

Alignments that demonstrate the interesting element-based *SpTrf* cDNA sequence structure is superficially consistent with and suggestive of extensive alternative splicing similar to that documented for *DSCAM* (22). However, when genomic DNA (gDNA) from three sea urchins is digested with restriction enzymes, used in Southern blots, and analyzed with probes from the 5′ and 3′ ends of cDNA templates, both probes hybridize to bands of 1.5–2 kB, which are similar in size to the mRNA sequences (Figure 3A) (6). This prediction of a small gene size does not fit with the *DSCAM* gene structure of ~100 exons and correlates with results from a search of the initial assembly of the sea urchin genome sequence (9/2003) that shows *SpTrf* genes of less than 2 kB with two exons (Figure 3B). Alternative splicing to generate the cDNA sequence diversity is impossible for two exons, and no cryptic splice sites are present in the genes that might generate unexpected splicing patterns (23). Because the *SpTrf* genes are small, they could be amplified by PCR from gDNA and sequenced, and all show the same basic structure of two exons (15). Comparisons among 121 genes of unique sequence (of 171 sequenced gene amplicons) show significant sequence diversity. Although the first exon encodes a relatively conserved hydrophobic leader, the second exon is highly diverse with regard to both size and sequence and encodes the mature protein with mosaic element patterns corresponding to those characterized in the cDNAs (Figure 2A) (15). When the coding regions of the genes are aligned using the cDNA-based alignment parameters according to Terwilliger et al. (6), the first four elements in the second exon are not defined by the insertion of artificial gaps (Figure 2A). Furthermore, the edges of the elements and the edges of the repeats do not correspond. Consequently, an alternative alignment that matches the edges of the repeats with the edges of the elements, where possible, resulted in the “repeat based” alignment for both genes and cDNAs (Figure 2B). The repeat-based alignment collapses some repeats, identifies the type 6 repeat, and increases the number of possible elements to 27 although it shortens the overall length of the alignment. As expected, the intron sequences are more diverse than the exons, although comparisons among the introns suggest five types that are usually, but not always, associated with a specific element pattern in the second exon (15). Alignments of the genes reveal several surprising results besides the presence of elements and repeats. Comparisons among gene sequences from different sea urchins show that no full-length gene sequence is shared among animals, but that sequences of individual elements, which have different sequence variants, can be shared among genes from individual animals and among different animals (Figure 3C). The *SpTrf* genes are unique and highly unusual based on their significant sequence diversity that is derived from the element-based structure of the second exon in addition to sequence variations in many of the elements.

## *HeTrf* Genes are also Structured with Elements

The sea urchin *H. erythrogramma* is local to Australia and the southern hemisphere and is morphologically similar to *S. purpuratus*. They are about the same size, are generally purple, and have similar types of coelomocytes in the coelomic fluid (CF) (24). Although, their life histories are quite different—*S. purpuratus* is an indirect developer with larvae that feed in the zooplankton prior to undergoing metamorphosis to a juvenile sea urchin, whereas *H. erythrogramma* skips the larval stage and develops directly from an embryo to a juvenile—both species have *Trf* gene families (21). The *HeTrf* cDNA sequences are 68–74% identical to the *SpTrf* cDNA sequences, tend to be shorter, and have 31 elements arranged into 29 different element patterns that are different from those in the *SpTrf* cDNAs and genes. The *HeTrf* genes also have two exons, although the intron has large variations in length. There are four types of imperfect tandem and interspersed repeats that are similar to four of the six repeats in the *SpTrf* sequences, although the copy numbers and positions of the repeats within the genes are different. Codons under positive selection for diversification [for methods, see references in Ref. (21)] are positioned throughout the sequences for the *HeTrf* genes but tend to be located within the first 200 codons in the *SpTrf* genes. These two *Trf* gene families are clearly homologous but the two families separate into different clades in phylogenetic analyses suggesting diverging evolutionary histories likely based on different sets of pathogens that the two species face not only as adults but also during the larval phase of *S. purpuratus*, which is absent in *H. erythrogramma*.

## Evolutionary History of the *SpTrf* Genes Estimated from the Type 1 Repeat Diversity

The varieties of repeats in the *SpTrf* genes are a notable and unusual attribute of the second exon. The five types of interspersed repeats positioned toward the 3′ end of the second exon are present in complex patterns that are repeated two or three times depending on the alignment (Figure 2) (25). The tandem type 1 repeats that are present in two to four copies are positioned toward the 5′ end of the second exon and show imperfect sequence matches in addition to mosaic patterns that vary among genes (Figure 2). A computational evaluation of the type 1 repeats and their phylogenetic clustering into four clades demonstrated that clade membership correlates with their position in the second exon and defines the correct position of the repeats when two or three are present rather than four (25). When two type 1 repeats are present in a gene, they are always the first and fourth repeat, and when three repeats are present, they are always the first, second, and fourth repeat (Figure 2B). Sequence variations among the type 1 repeats may be the outcome of duplication, deletion, and recombination of two theoretical ancestral type 1 repeat sequences that are based on a computational prediction from extant sequences. This led to questions of whether recombination hot spots could be identified within the genes, which was underpinned by observations that sequences of adjacent regions did not match among different genes. For example, these included (i) the sequence of the 5′ UTR relative to the adjoining first exon, (ii) the sequence of the 5′ end vs. the 3′ end of the genes, and (iii) the 5′ vs. 3′ ends of some elements irrespective of whether they correspond to repeats (25). Predictions strongly suggest significant recombination between the two ends of the second exon, between adjacent elements, and within larger elements, with no clear hot spots of recombination (Table 1). Furthermore, the frequency of predicted recombination within the second exon is similar to results for the well-known somatic recombination that occurs among the variable and joining segments of the T cell receptor and is very different from the lack of recombination between the two ends of the sea urchin histone *H3* gene. Molecular clock analysis of the *SpTrf* genes indicates that the genes are young (26) and about the same age as the species (27), which is in agreement with the generally accepted concept that immune genes encoding proteins that interact with the environment and/or pathogens are under pressure to diversify and show swift evolution [reviewed in Ref. (1, 2)]. The occurrence of recombination throughout the *SpTrf* gene family is likely to be much greater than suggested by shared and unshared element sequences and may perhaps be driven by the clustered nature of the genes (2, 28, 29) (see below).

**Table 1:** Recombination is predicted throughout the *SpTrf* gene sequences.^a^

	L	IntA	IntB	1	6	6a^b^	6b	26	27	27a	27b	*H3*.1^c^	*H3*.2	*TcRV*^d^	*TcRJ*
L		***	***	**	**	▫	▫	***	***	▫	▫	▫	▫	▫	▫
IntA	**		***	**	***	▫	▫	***	***	▫	▫	▫	▫	▫	▫
IntB	**	**		ns	***	▫	▫	***	***	▫	▫	▫	▫	▫	▫
1	**	**	*		***	▫	▫	***	***	▫	▫	▫	▫	▫	▫
6	*	**	**	**		▫	▫	***	***	▫	▫	▫	▫	▫	▫
6a	▫	▫	▫	▫	▫		*	▫	▫	▫	▫	▫	▫	▫	▫
6b	▫	▫	▫	▫	▫	*		▫	▫	▫	▫	▫	▫	▫	▫
26	**	**	**	**	***	▫	▫		***	▫	▫	▫	▫	▫	▫
27	**	**	**	**	***	▫	▫	*		▫	▫	▫	▫	▫	▫
27a	▫	▫	▫	▫	▫	▫	▫	▫	▫		*	▫	▫	▫	▫
27b	▫	▫	▫	▫	▫	▫	▫	▫	▫	**		▫	▫	▫	▫
*H3*.1	▫	▫	▫	▫	▫	▫	▫	▫	▫	▫	▫		ns	▫	▫
*H3*.2	▫	▫	▫	▫	▫	▫	▫	▫	▫	▫	▫	ns		▫	▫
*TcRV*	▫	▫	▫	▫	▫	▫	▫	▫	▫	▫	▫	▫	▫		***
*TcRJ*	▫	▫	▫	▫	▫	▫	▫	▫	▫	▫	▫	▫	▫	**	

*^a^Recombination within and among elements is based on results from the incongruence length difference test shown above the diagonal and the incongruence permutation test shown below. This table is modified from Ref. (25)*.

*^b^For some individual elements, comparisons are carried out between the 5′ end (a) and the 3′ end (b). Element numbers are based on the repeat-based alignment (see Figure 2B)*.

*^c^Evaluation of the sea urchin histone *H3* gene sequence employed two regions of the gene sequence, *H3*.1 and *H3*.2, which were of similar size to the average element size in the SpTransformer (*SpTrf)* sequences*.

^d^Evaluation of the TcR employed the variable region (*TcRV*) and the joining region (*TcRJ*) (*p < 0.05, **p < 0.01, ***p < 0.001; ns, not significant; L, leader; Int, intron; ▫, not done).

## The *SpTrf* mRNAs are Likely Edited

A surprising result from the *SpTrf* cDNA sequences reported by Terwilliger et al. (11) is that only about half (306 of 608) encode full-length proteins, whereas the rest have frameshifts leading to missense sequence and early stop codons or have a single nucleotide change that inserts an early stop codon at a particular position in element 13 (Figure 2A). Similarly, point mutations, indels, missense sequence, and early stop codons are also present in about 10% (11 of 112) of the *HeTrf* cDNA sequences from *H. erythrogramma* (21). In striking comparison, all but one of the 198 *SpTrf* gene sequences [171 amplified from gDNA from three sea urchins, 12 amplified from clones in the small insert bacterial artificial chromosome (BAC) library (15), and 15 assembled from BAC inserts (29)] have perfect open reading frames. The unusual difference of perfect vs. altered reading frames in the genes vs. the cDNAs, respectively, is an outcome of comparisons between genes and cDNAs from individual animals (30). Very few of the genes match identically to the cDNA sequences from individual sea urchins, but more noteworthy are the differences between the sequences of the genes and cDNAs of the same element pattern. The comparison shows that 30% of the nucleotide differences are a cytidine in the gene and a uracil at the same position in the cDNA, which is consistent with cytidine deaminase activity (30). Other changes in the cDNAs, such as the indels, may be the outcome of low fidelity RNA polymerases, such as polymerase μ. Genes encoding several cytidine deaminases plus polymerase μ are present in the sea urchin genome sequence (31). These results suggest editing of the *SpTrf* mRNAs, which, although quite unexpected, could have the disadvantage of yielding transcripts that encode non-functional proteins, but also the advantage of expanding the diversity of the proteins produced in response to immune challenge irrespective of whether the editing may be random, directed, or both.

**Figure 4:**
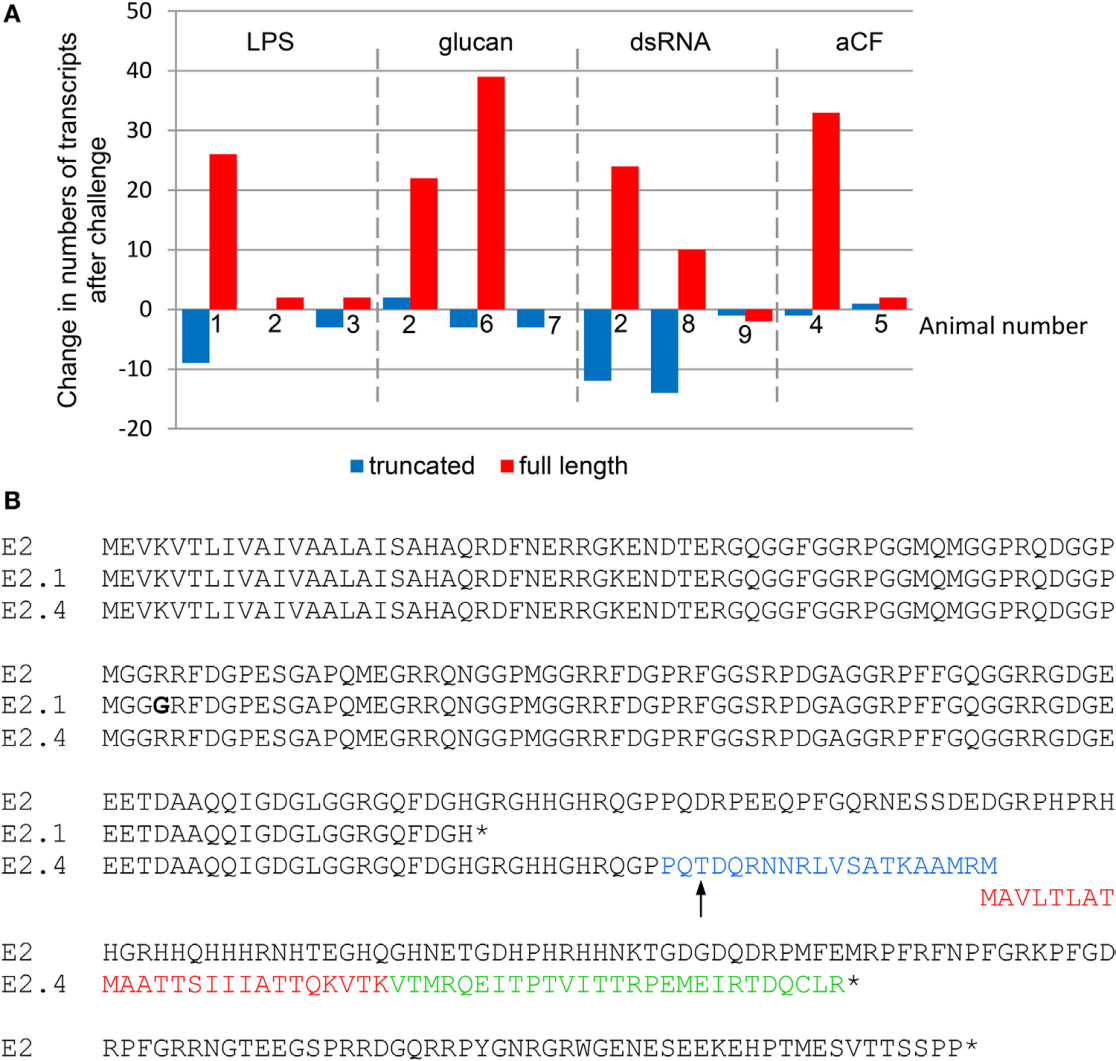
Immune challenge decreases edited *SpTransformer* (*SpTrf)* mRNAs. **(A)** The change in transcripts encoding truncated (edited) vs. full-length sequences with regard to immune challenge with lipopolysaccharide (LPS), β-1,3-glucan (glucan), double stranded RNA (dsRNA), or sham injection (aCF; artificial coelomic fluid) relative to pre-challenge transcript numbers is shown for nine animals based on cDNA sequences reported previously (11). Animal 2 received separate challenges from all pathogen-associated molecular patterns. Amplicons from RT-PCR for animals that received LPS that were used for this analysis are shown in Figure 1C. Bars below 0 indicate fewer transcripts after challenge and bars above 0 indicate more. Missing bars indicate no change. This figure is modified from Ref. (33). **(B)** An alignment of deduced amino acid sequences from a full-length E2 protein and two truncated E2 proteins shows mismatches, frameshifts, and early stops. The SpTrf protein with an E2 element pattern is a full-length protein encoded by cDNA clone Sp0016 [GenBank accession number DQ183104.1 (6)]. In some cDNA sequences denoted E2.1, the sequence is edited at a specific glycine codon to a stop that is not encoded by the gene. The E2.1 truncated sequence is encoded by cDNA clone 1-1539 [GenBank accession number EF066308.1 (11)] and prior to the early stop is not identical to the E2 sequence used in the alignment (bold glycine is indicated). The E2.4 element pattern is an edited mRNA and encodes a truncated protein with missense sequence (cDNA clone 8-2415; GenBank accession number EF065834.1 (11)). The point of the frameshift is indicated with an arrow, which is followed by missense sequences that have been identified by proteomic methods (blue and red text) (32). Additional missense sequence in E2.4 is shown in green followed by an early stop codon. The alignment was done with BioEdit (34) and modified by hand. Stop codons are indicated by the (*).

The identification of RNA editing of both *SpTrf* and *HeTrf* transcripts resulting in indels and frameshifts led to an initial assumption that these mRNAs would be recycled and not transcribed. However, predicted missense sequences from edited cDNA sequences with frame shifts are present in the SpTrf proteins isolated from the CF, indicating that the edited mRNAs are translated (32). This is noteworthy because the frequency of edited vs. non-edited mRNAs changes relative to immune challenge. Edited *SpTrf* mRNAs encoding truncated proteins including some with missense sequence tend to be present more often in coelomocytes from IQ sea urchins prior to immune challenge, whereas mRNAs that are not edited and encode full-length proteins tend to increase in coelomocytes responding to immune challenge (Figure 4A) (11, 33). This change is detected in many sequence versions of the cDNAs but is most easily identified in those that encode the *E2* element pattern (494 of 608 cDNAs) of which 57% have a nucleotide change in element 13 that changes a glycine codon to an early stop (Figure 2A). This single edit results in truncated proteins that are missing the histidine-rich region and are defined as the *E2.1* element pattern (11). Edits to the *E2* mRNA can also insert indels that induce frameshifts, such as the E2.4 sequence that has missense sequence and an early stop (Figure 4B). An alignment of deduced protein sequences with the E2 element pattern illustrates the position of the common RNA-editing event that produces the E2.1 truncated protein (Figure 4B). RNA editing that deletes the histidine-rich region of proteins is consistent with difficulties in isolating many SpTrf proteins by nickel affinity prior to challenge (33). Speculation on the underlying basis for the change in editing relative to an immune response suggests that at least the E2.1-truncated proteins may have broad immuno-surveillance functions, whereas the full-length proteins may be more targeted to particular pathogens (3) (see below).

## *SpTrf* Gene Family Size and Structure

**Figure 5:**
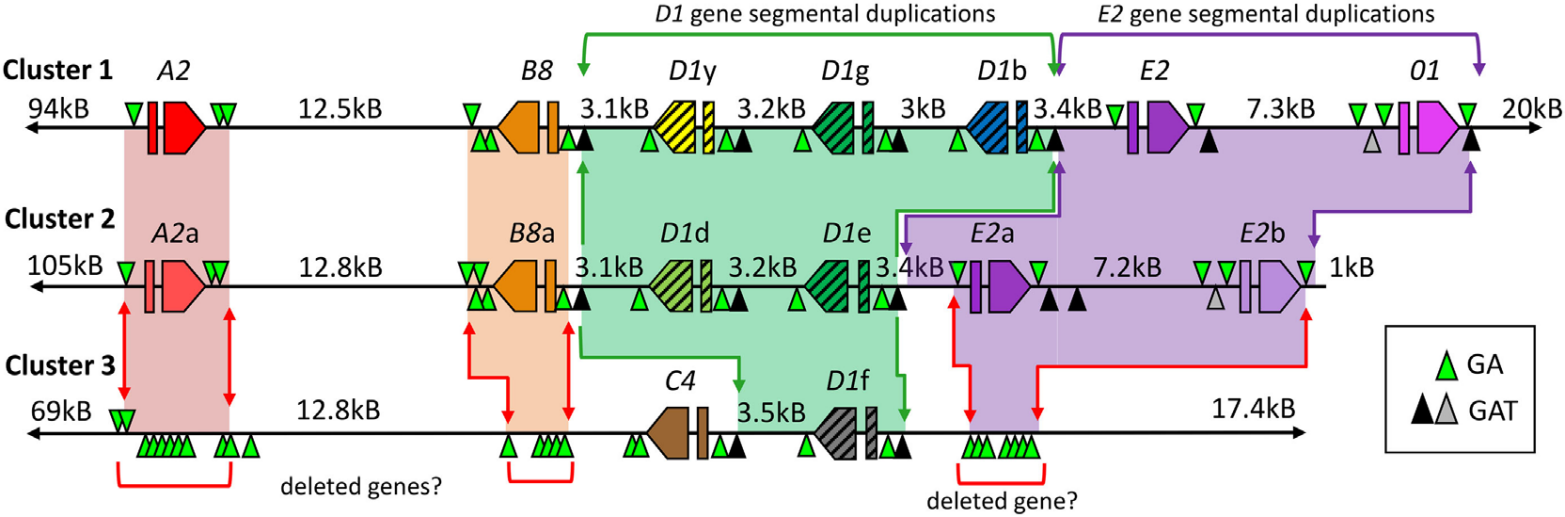
Three clusters of *SpTransformer* (*SpTrf*) genes are present in the *Strongylocentrotus purpuratus* genome. The three clusters of genes are likely located at two loci within the genome. See Figure 3B for an illustration of the standard gene structure. Clusters 1 and 2 are likely allelic based on matches in the flanking regions outside of the gene clusters, even though the numbers of genes within the loci do not match. Genes are labeled by element pattern; however, those with the same element pattern are not necessarily of identical sequence. All genes are flanked by GA short tandem repeats (STRs) and may be the basis of deleted regions (red arrows), including genes, in Cluster 3 that are indicated by regions of GA STRs that are as long as 3 kB. Segmental duplications including *D1* genes (green shading and green arrows) and *E2* genes (purple shading and purple arrows) are flanked by GAT STRs (black triangles indicate >35 repeats, gray triangles indicate 4–17 repeats). Red and orange shading indicate likely alleles in Clusters 1 and 2. Regions of missing or deleted genes in Cluster 3 are indicated by red brackets. This figure is modified from Ref. (29).

The extraordinary diversity of 121 (~71%) unique sequences of 171 amplified *SpTrf* genes from three *S. purpuratus* sea urchins predicts that the gene family is likely large. Detailed analysis plus three different approaches for estimating the gene family size predicted ~50 ± 10 *SpTrf* genes per genome [reviewed in Ref. (16)]. In stark contrast to this estimate, only six genes are assembled in the sea urchin genome sequence. This lack of correlation may be the outcome of significant artifacts in genome assembly for genes with shared sequences that are tightly linked and associated with repeats (35). The apparent underestimation of the *SpTrf* gene family in the assembled genome sequence may be the result of assembling similar genes into hybrid sequences that do not actually exist in the real genome (2, 28, 29). Finding the correct structure and sequence of the *SpTrf* gene family led to a screen of the sea urchin gDNA BAC library followed by insert sequencing, assembly, and annotation that identified three clusters for a total of 15 *SpTrf* genes (Figure 5) (28, 29). Although 15 genes are many fewer than predicted, it is consistent with 18 genes predicted from the genome sequence traces available prior to assembly. Although it is possible that the *SpTrf* gene clusters may be unstable in BAC clones (see below), it is also feasible that the animal that provided gDNA for genome sequencing may have had a particularly small *SpTrf* gene family.

The clusters of *SpTrf* genes in the sea urchin genome sequence are positioned on both the positive and negative DNA strands in mixtures of genes with different element patterns that show significant sequence diversity within the clusters (Figure 5) (29). The genes are linked as tightly as 3 kB, although the flanking genes in Clusters 1 and 2 are positioned much farther from their nearest neighbor. All genes are flanked by short tandem repeats (STRs) of GA sequences. Moreover, all six of the *D1* genes and two of three of the *E2* genes are positioned within segmental duplications that are flanked by GAT STRs. The segments harboring the six *D1* genes are highly similar as are those with the three *E2* genes in addition to the *01* gene in Cluster 1 (Figure 5) (28, 29). The long flanking regions on either side of Clusters 1 and 2 are very similar indicating that these two clusters are likely allelic even though the numbers of genes and their element patterns do not match. Clusters 1 and 2 are most similar to the *SpTrf* gene cluster on scaffold 125 of the sea urchin genome sequence; however, the genes on the scaffold appear to be hybrid sequences of both allelic clusters (and consequently are artificial sequences) and do not include the *01* gene in Cluster 1. Hybrid gene sequences are predicted based on assembly approaches that use sequence reads from both alleles at a locus, compounded by efforts to avoid assembling both alleles in what would appear as tandem gene duplicates. Cluster 3 is quite different from Clusters 1 and 2 and is positioned at a different locus because the flanking regions do not match those of Clusters 1 and 2 (Figure 5). The two genes in Cluster 3 are positioned in the same orientation and are both surrounded by GA STRs, but only the *D1*f gene is positioned within a segmental duplication flanked by GAT STRs that shows sequence similarity to the *D1* duplications in the other two clusters. Outside of the two *SpTrf* genes in Cluster 3 are flanking sequences with GA STRs of about 3 kB that are positioned at locations of ~3 kB and ~12 kB from the two genes, which match the locations of genes in the other two clusters. Speculations on the positions and functions of the STRs in the *SpTrf* gene clusters suggest that the GAT STRs may drive segmental duplications of regions that include the *D1* and *E2* genes (29). Sequence similarities among regions between the GA STRs that include the genes suggest that they may drive gene duplications (28). However, the size and locations of GA STRs flanking the genes in Cluster 3 are also consistent with gene deletions (2, 29). The non-matching allelic loci in Clusters 1 and 2 that include both different numbers of genes and variations in the element patterns in the second exon among the genes is consistent with the concept of genomic instability that may be based on shared sequences, shared repeats, and the association with many STRs within the clusters of this gene family [(2) and see below].

Although the concept of genomic instability intuitively seems lethal in that it could compromise both coding and regulatory regions, there can be advantages to genomic instability in localized and restricted regions. The advantage of small, tightly linked genes with shared patches of sequence, nearly identical segmental duplications, and tightly associated STRs, is that these attributes are likely essential for the sequence diversification of the *SpTrf* gene family (2). Rapid diversification is common for many innate immune genes that are under pathogen pressure and must keep pace in the arms race for host survival (1). This is consistent with swift changes in the members of the *SpTrf* gene family with the advantage of driving broad diversity of the expressed proteins (33, 36) that may be essential for interactions with the populations of microbial and other pathogens in the ocean that are simultaneously under selection for virulence to improve invasion, proliferation, and survival. A characteristic of many clustered genes that encode proteins with activities for interacting in some way with the environment such as pathogen recognition receptors or odorant or taste receptors (among others) is that although the genes tend to change rapidly, the diversification process generates pseudogenes. For example, 25% of the 253 clustered *SpTLR* genes in the sea urchin genome sequence are pseudogenes (37), and 54% of the clustered human odorant receptor gene superfamily are pseudogenes (38). Mechanisms for correcting the reading frames in *SpTrf* pseudogenes have been speculated upon and may be an aspect of gene sequence diversification mechanisms, which are related to tight gene clustering (2, 28). Crossing over and gene conversion are enhanced in regions of the *Arabidopsis* genome that contain shared sequences, such as the disease resistance gene family (39). This process may also function for the *SpTrf* gene clusters based on the abundant shared sequences within and among the clusters. However, there must be some level of balance for gene conversion that would correct reading frames but with controls to block sequence homogenization among multiple linked family members. Homogenization of gene sequences within clusters would be disadvantageous in the arms race against pathogens. Hence, the conversion process that runs through a gene may be initiated by sequences shared among genes, but that progression to tightly linked genes may be limited by the presence of the GA STRs that surround all genes (28). This is consistent with increased sequence diversity in intergenic regions (excluding intergenic regions that are part of segmental duplications). However, a single *SpTrf* pseudogene that has been identified from 198 sequenced *SpTrf* genes has a deletion that alters the reading frame and is unusual because it is intronless and may be a retroposon. Possibilities as to why a retroposon may show a frameshift could be that it may not be expressed if it is not associated with a regulatory region and, therefore, may not be under pathogen pressure to maintain the ORF. Furthermore, if it was retro transposed into the genome in isolation away from clustered *SpTrf* genes, the theoretical mechanisms for diversification and reading frame corrections may not extend to isolated genes. The overall genomic instability predicted for the *SpTrf* gene family that is based on multiple types of repeats within and surrounding the clustered genes is consistent with the observation of differences in the repertoire of genes in the *SpTrf* family among individual sea urchins (29). Ongoing diversification of the *SpTrf* genes and the advantages of this process for host protection against pathogens require the input of new genes to the family as others are modified and/or deleted, and fits a description of swift evolution and the birth–death or duplication–deletion concept for duplicated genes (40).

## Diversity of the SpTrf Proteins

**Figure 6:**
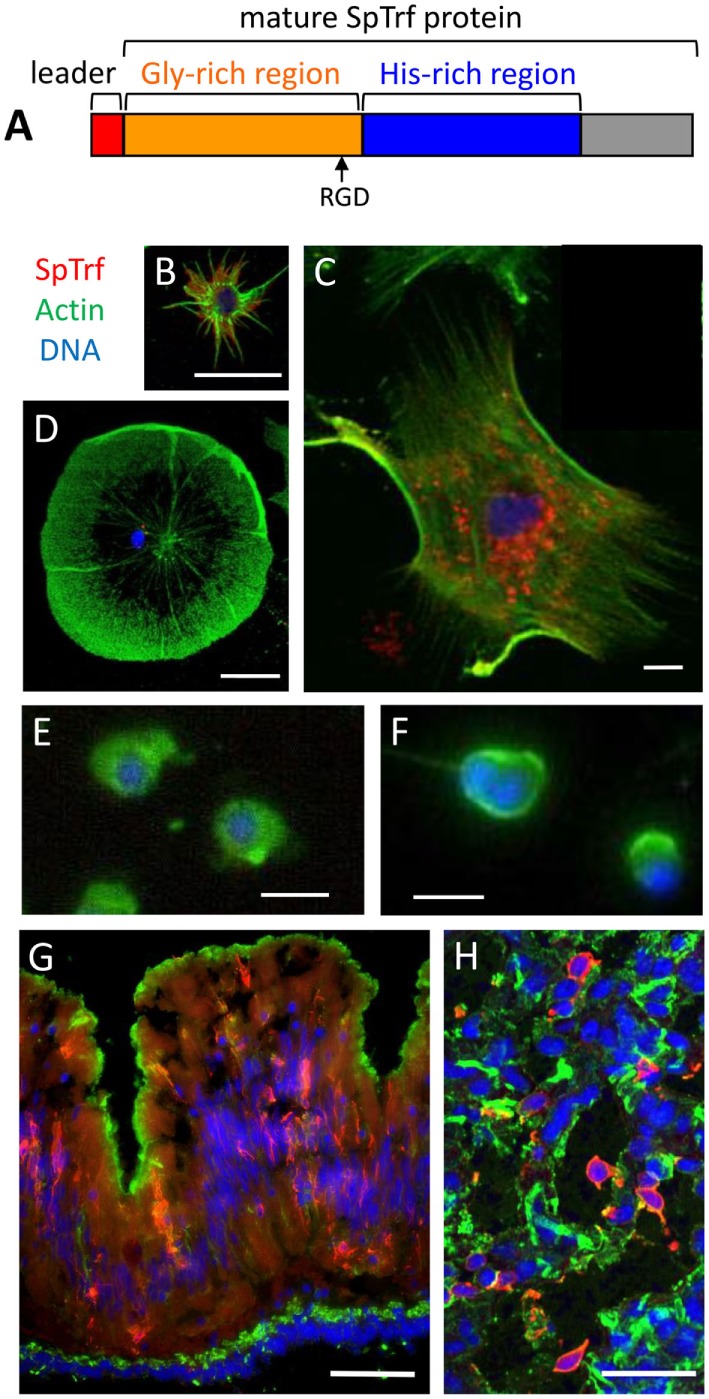
The phagocyte subpopulation of coelomocytes expresses the SpTransformer (SpTrf) proteins. **(A)** The standard SpTrf protein structure has an N-terminal leader (red), a glycine-rich region (orange), a histidine-rich region (blue), and a C-terminal region (gray). This figure is reprinted from Ref. (1). **(B)** A small phagocyte has SpTrf proteins within the cell and on the cell surface. **(C)** A large polygonal phagocyte has SpTrf proteins in small vesicles surrounding the nucleus. **(D)** A few discoidal phagocytes have a few, perinuclear vesicles containing SpTrf proteins. **(E)** Red spherule cells and **(F)** vibratile cells do not express SpTrf proteins. **(G)** A cross section of gut shows SpTrf^+^ cells within the columnar epithelium that are likely coelomocytes. The gut lumen is at the top of the image and the coelomic cavity is toward the bottom. **(H)** Numerous SpTrf^+^ cells are present within the axial organ, and are likely coelomocytes. Fluorescence microscopy was used to generate images **(B,D–G)**, and confocal microscopy was used for **(C,H)**. Images **(B,C)** were contributed by A. J. Majeske. **(D–F)** were reproduced from Ref. (41) with permission. Copyright 2014. The American Association of Immunologists, Inc. **(F,G)** were reprinted from Ref. (42). Scale bars are 10 µm for **(B–F)** and are 100 µm for **(G,H)**.

The rapid onset and increase in *SpTrf* gene expression in sea urchins upon immune challenge from microbes or PAMPs (8, 9, 11), the sequence diversity of the genes, and messages (6, 11, 15, 28, 29) in addition to putative mRNA editing (30) suggest that the encoded proteins are highly diverse and likely have immunological functions. The deduced structure of the SpTrf proteins indicates a hydrophobic leader and a mature protein of variable sizes that includes a glycine-rich region near the N-terminus with an arginine–glycine–aspartic acid (RGD) motif near the middle of most proteins suggestive of integrin binding, followed by a histidine-rich region, and a C-terminal region (Figure 6A). The deduced sizes and sequences of the glycine-rich and the histidine-rich regions are highly variable based on the presence and absence of elements and the sequence variability within elements in the genes and messages (see Figure 2). The HeTrf proteins from *H. erythrogramma* have a similar structure including a C-terminal histidine-rich region with poly-histidine patches that vary from 6 to 13 histidines (21), which is more histidines than that have been identified in most of the SpTrf proteins. Only a few of the HeTrf proteins have an RGD motif whereas it is present in most of the SpTrf proteins. The HeTrf proteins are composed of subsets of 26 possible elements and have four types of imperfect repeats that are positioned in both tandem and interspersed patterns. The sequences of both the elements and the repeats in HeTrf proteins are somewhat similar to those in SpTrf proteins, although the organization is different. These two homologous gene families encode proteins predicted to have similar anti-pathogen functions; however, their characteristics are not identical (21).

## SpTrf Proteins are Expressed in a Subset of Phagocytes

There are four major morphotypes of coelomocytes in *S. purpuratus* that include phagocytes, red and colorless spherule cells, and vibratile cells (24), and only some of the phagocyte class of coelomocytes express the SpTrf proteins (41, 43). Surprisingly, the cells with the highest SpTrf expression are the small phagocytes in which the proteins are localized to cytoplasmic vesicles and the cell surface (Figure 6B). Some of the large phagocytes have SpTrf proteins localized to vesicles surrounding the nucleus but the proteins are never found on the cell surface (Figures 6C,D). The red spherule cells and the vibratile cells are consistently negative for SpTrf expression (Figures 6E,F). The expression patterns for HeTrf proteins in *H. erythrogramma* are similar to patterns of the SpTrf proteins, are localized to perinuclear vesicles, and are on the surface of some phagocytes (21). Analysis of the SpTrf protein expression patterns has benefited from the use of IQ sea urchins that tend to have decreased numbers of coelomocytes in the CF (43). When IQ sea urchins are challenged with LPS, there is a twofold increase in the total number of coelomocytes in the CF after 24 h and a 10-fold increase in the SpTrf^+^ cells in the CF after 48–96 h (36, 43). Of those increased numbers of cells in the CF, the small phagocytes show a significant increase including more cells that express SpTrf proteins. In parallel, the percentage of polygonal phagocytes in the CF does not change in response to LPS; however, these cells tend to increase expression of the SpTrf proteins. These results may be interpreted as the production and secretion of SpTrf proteins from the polygonal phagocytes and the secretion plus acquisition of SpTrf proteins onto the surface of small phagocytes.

**Figure 7:**
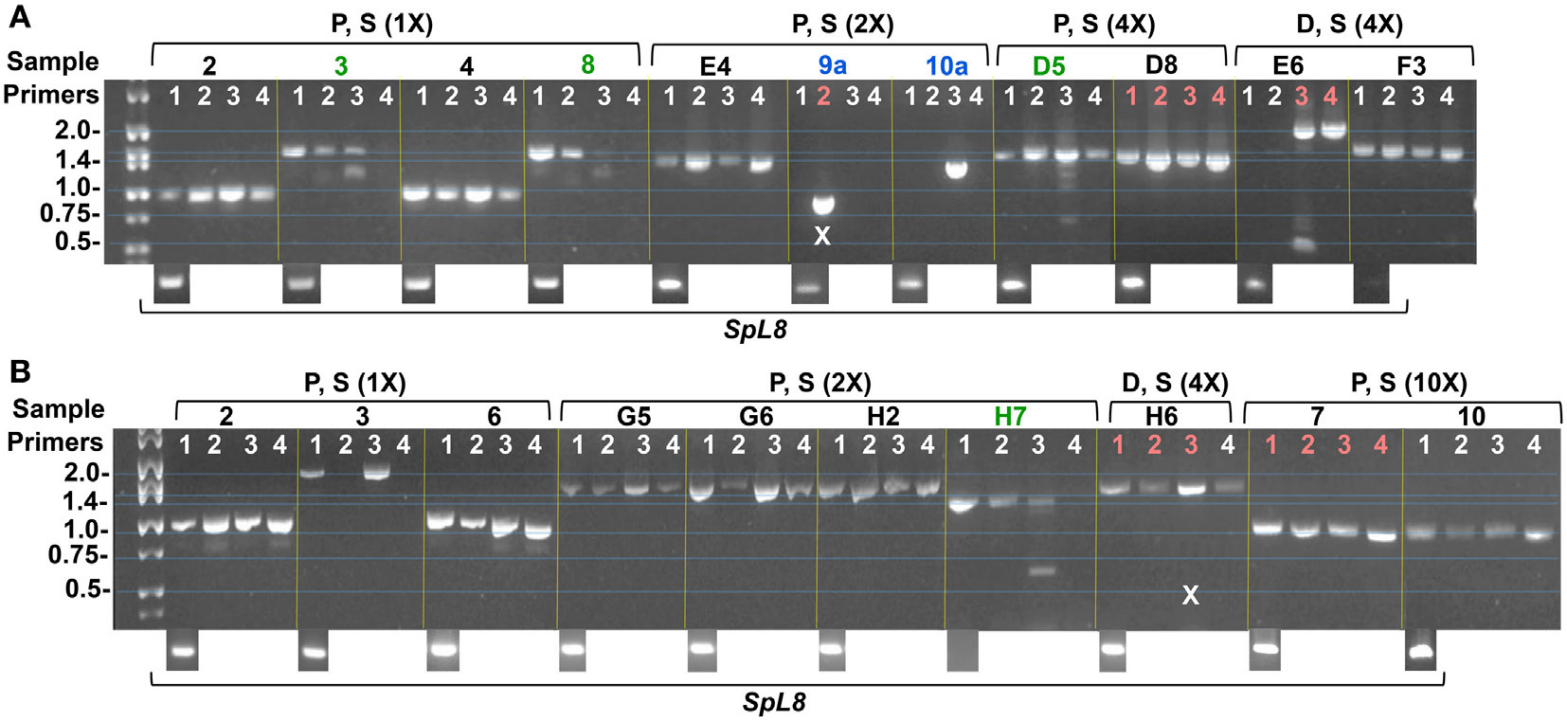
Amplicons from single phagocyte indicate expression of single *SpTransformer* (*SpTrf)* genes in single cells. Coelomocytes were collected from two sea urchins **(A,B)**, fractionated by Percoll density gradient into fractions of polygonal plus small phagocytes (P, S), and discoidal plus small phagocytes (D, S). Fractions were diluted to an estimate of 1 cell/μl followed by further dilutions of 2×, 4×, and 10× to ensure 1 cell/sample. Samples were first tested by nested RT-PCR using primers for *SpL8* (shown at the bottom of the images) that encodes the sea urchin homolog of protein 8 from the human large ribosomal subunit, and indicates samples that contain a cell. Samples with cells were evaluated for *SpTrf* transcripts by nested RT-PCR using four pairs of primers (1–4) on each sample that would amplify different sequence versions of *SpTrf* cDNAs. Green sample numbers indicate multiple bands amplified by the fours primer pairs. Blue sample numbers indicate a single amplicon for a single pair of primers. Samples indicated in red were chosen for sequencing. X indicates failed or ambiguous sequence results. This figure is reproduced from Ref. (41) with permission. Copyright 2014. The American Association of Immunologists, Inc.

The swift pattern of *SpTrf* gene expression in phagocytes responding to immune challenge or injury can be imagined conceptually as the expression of as many of the *SpTrf* genes as quickly as possible and production of as many of the SpTrf proteins as appropriate to control or eliminate the detected pathogen. This would be advantageous in responding to infections and to protect the host from being overwhelmed by and succumbing to a pathogen. Surprisingly, when single phagocytes are evaluated for *SpTrf* transcripts, not only do most of the individual cells yield *SpTrf* amplicons of the same size (Figure 7) but the amplicon sequences from single cells are the same (41). This implies that one gene from the *SpTrf* family is expressed per individual phagocyte. Because sea urchins show a significant increase in messages (11) and SpTrf protein arrays (33, 36) in response to immune challenge, this swift response was considered feasible only if multiple *SpTrf* genes were expressed per phagocyte. Consequently, expression of a single *SpTrf* gene per phagocyte was an unexpected outcome. The mechanism for how this is regulated including expression of one gene and suppression of all the others, perhaps in response to the particular pathogen, is not known.

## SpTrf Expression in Adult and Larval Sea Urchin Tissues

In addition to expression in the phagocyte class of coelomocytes in adult sea urchins, SpTrf protein expression is also associated with non-immune tissues. Some of the cells within the columnar epithelium of the gut express SpTrf proteins (Figure 6G) (42), and similarly, the HeTrf proteins are localized to membranes of transport vesicles and the plasma membrane in gut associated amebocytes (or phagocytes) (44). In addition to the gut epithelium, SpTrf proteins are also expressed in the pharynx, esophagus, and gonads (42). It is noteworthy that expression of the SpTrf proteins also occurs in the axial organ (Figure 6H), which shows increased expression after immune challenge. Although SpTrf proteins in sea urchin larvae have not been reported, *SpTrf* gene expression is restricted to a subset of blastocoelar cells that are localized in the blastocoel, extend filopodia across the blastocoel, form syncytia (45), and function as the primary larval phagocytes and act in host protection (46). The larval blastocoelar cells appear to be the functional equivalent of the large phagocytes in adult sea urchins based on cellular morphology, localization in the body cavity, phagocytic activity, and syncytia formation (47). Given that the blastocoelar cells are the only cell type in larvae to express the *SpTrf* genes, it is likely that the SpTrf protein expression in adult tissues is similarly restricted to phagocytes.

## Diverse Arrays of SpTrf Proteins are Expressed in Response to Immune Challenge

**Figure 8:**
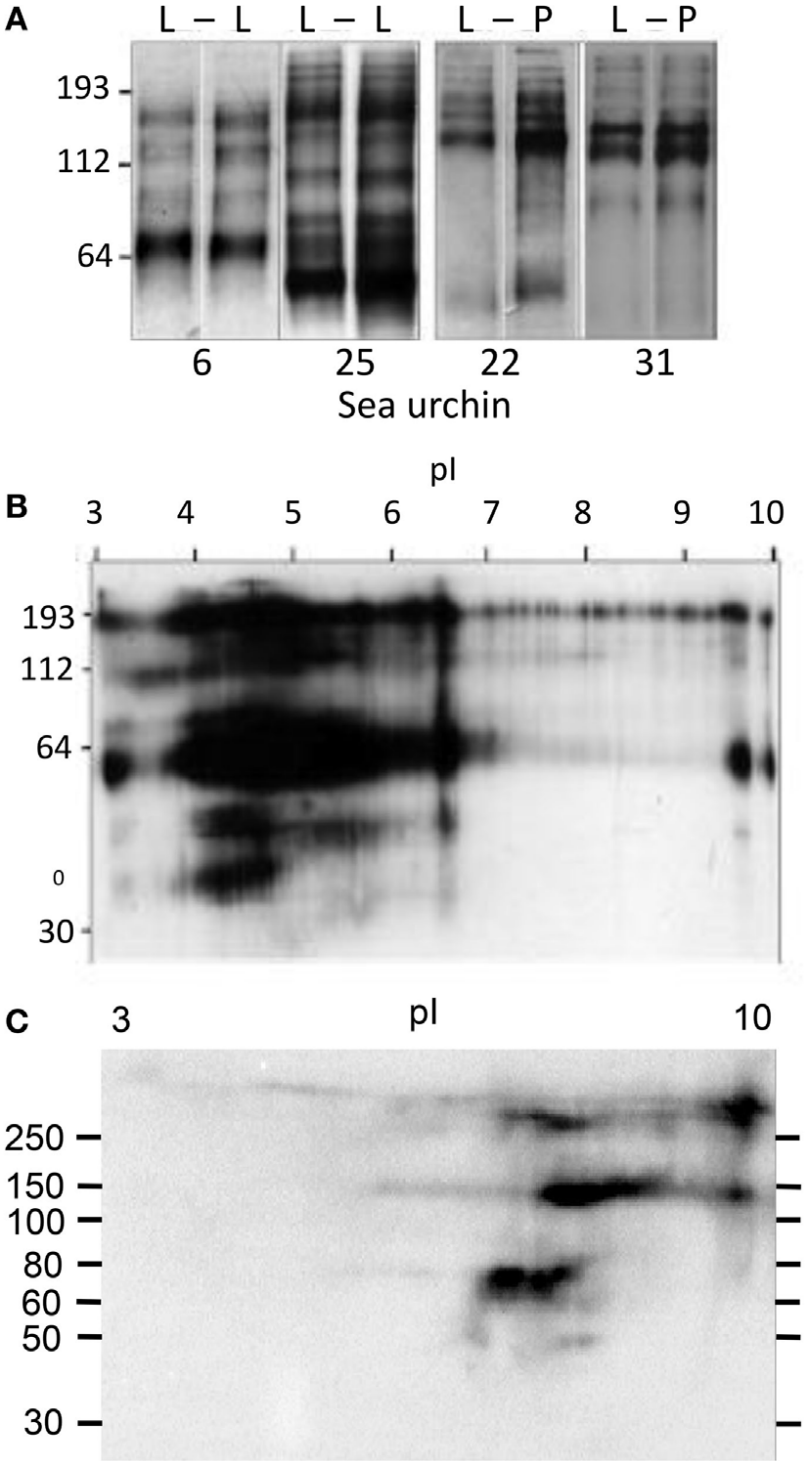
Diversity of the SpTransformer (SpTrf) proteins. **(A)** Sea urchins (#6 and #25) challenged with lipopolysaccharide (L) at 0 h (left lane) and after 320 h (right lane) were sampled for SpTrf diversity by Western blot 96 h after each injection. Two different sea urchins (#22 and 31) were challenged and analyzed similarly, but the second injection was peptidoglycan (P). Under both protocols, the SpTrf^+^ bands show diversity that varies with animal and challenge. This figure is reproduced from Ref. (36) with permission. Copyright 2009. The American Association of Immunologists, Inc. **(B)** Significant diversity in the SpTrf protein arrays from the coelomic fluid (CF) is illustrated by a 2D Western blot. The multiple horizontal protein trains suggest posttranslational modifications to proteins of the same molecular weight that alters the pI. Most of the proteins are present in the acidic range after isoelectric focusing. This figure is reproduced from Ref. (36) with permission. Copyright 2009. The American Association of Immunologists, Inc. **(C)** SpTrf proteins from the CF and isolated by nickel affinity show horizontal protein trains on a 2D Western blot as in **(B)**. However, nickel-isolated proteins tend to be basic, which is consistent with the preponderance of histidines in the C-terminal region of full-length proteins. This figure is reprinted from Ref. (33).

The predicted sizes of the SpTrf and HeTrf proteins from cDNA sequences range from ~4 kDa for the smallest truncated protein to 54 kDa for the largest full-length protein, and overall, the most common size range is 35–40 kDa (6, 11, 21). However, the actual average size of SpTrf and HeTrf proteins on Western blots is 65–80 kDa with much larger sizes of over 200 kDa, which is likely the result of multimerization (21, 36, 43). The patterns and sizes of bands on standard one-dimensional Western blots for SpTrf and HeTrf proteins are different among sea urchins and change differently in response to challenge, illustrating the level of diversity of these proteins within and among animals (Figure 8A) (21, 36). When the Trf proteins are isolated from the CF and evaluated by 2D Western blots after isoelectric focusing, the extent of protein diversity is displayed as arrays of spots of which many appear as horizontal trains of spots mostly in the acidic range suggesting variations in pI for proteins of the same molecular weight (Figure 8B) (21, 36). Full-length SpTrf proteins with sufficient numbers of histidines can be isolated by nickel affinity and they also appear on 2D Western blots as horizontal trains but are found in the basic region of the blot in accordance with the positive charge on the histidines (Figure 8C) (33). When nickel-isolated SpTrf protein arrays are compared among sea urchins, the arrays differ among animals and show differences in the numbers and intensities of the SpTrf spots. Furthermore, the SpTrf arrays among individual sea urchins change differently in response to a series of challenges from different species of bacteria (33). The extensive variations in the arrays of proteins in this family may be a combination of differences in numbers and varieties of genes in the *SpTrf* gene family among sea urchins plus the notion that changes in expression patterns may be tailored to the type of pathogenic challenge that is detected. This, in turn, suggests a detection system that has the ability to differentiate to some level among pathogens (36).

## Native SpTrf Proteins Bind Foreign Cells

**Figure 9:**
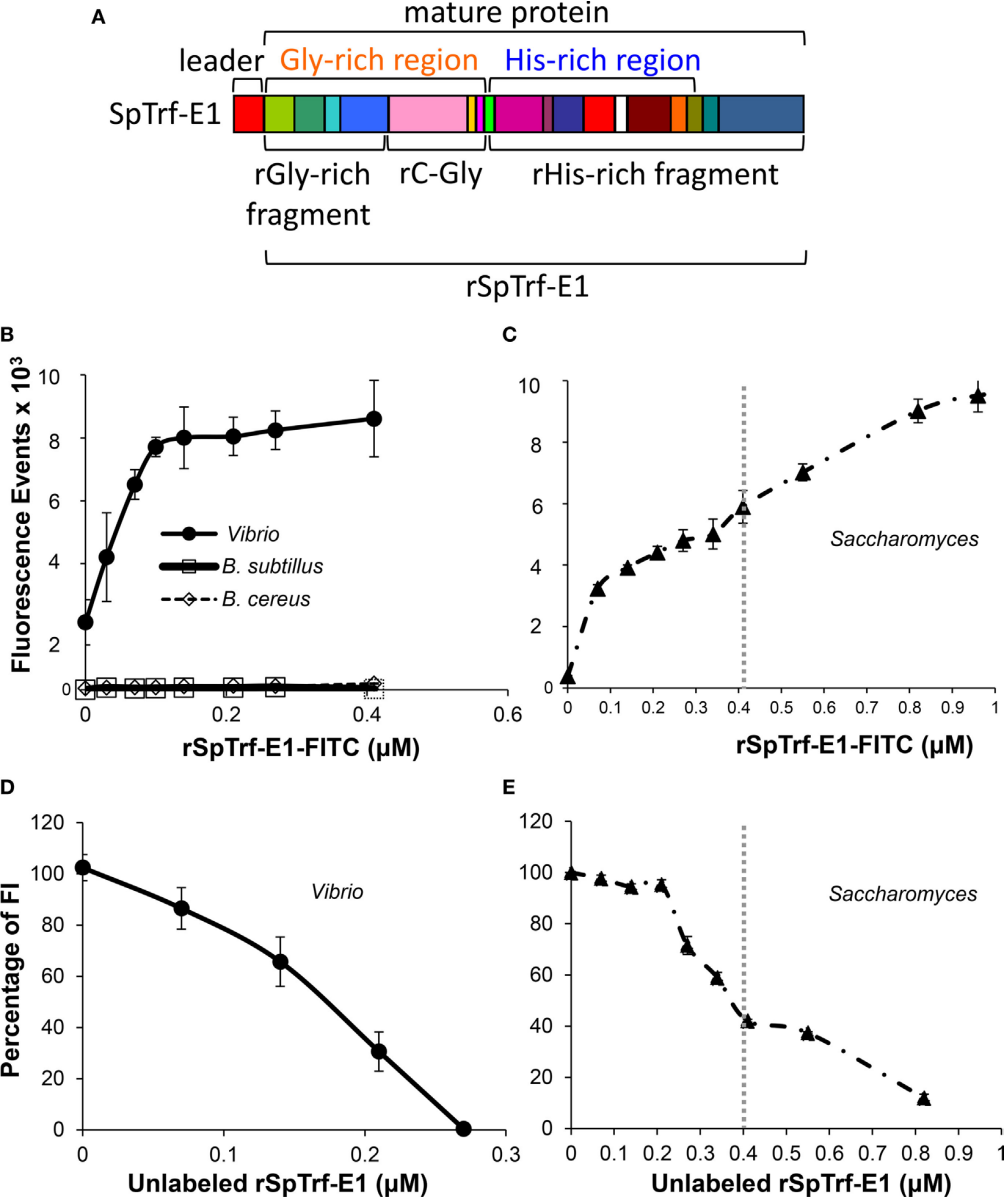
The deduced structure and element pattern of rSpTransformer-E1 (rSpTrf-E1) and binding characteristics toward bacteria and yeast. **(A)** The deduced, full-length rSpTrf-E1 sequence predicts a leader (indicated), which is likely cleaved from the mature protein, plus a glycine-rich region (orange text) and a histidine-rich region (blue text). This structure is consistent with the standard SpTransformer (SpTrf) structure (see Figure 6A). The mature rSpTrf-E1 protein is composed of a mosaic of elements (colored blocks) that are defined by gaps based on the “cDNA-based” alignment (see Figure 2A for matching element colors) and is defined as an E1 element pattern according to Terwilliger et al. (6). The full-length rSpTrf-E1 and the recombinant fragments are indicated. This figure is modified from Ref. (48). **(B)** rSpTrf-E1 labeled with FITC (rSpTrf-E1-FITC) shows saturable binding to *Vibrio diazotrophicus* based on the increasing fluorescence events by flow cytometry with increasing protein concentration. rSpTrf-E1-FITC does not bind to *Bacillus sutbtilis* or *B. cereus*. **(C)** rSpTrf-E1-FITC binds to *Saccharomyces cerevisiae* and shows two independent non-linear binding curves (separated by gray dotted vertical line) based on fluorescence events from flow cytometry. Both curves indicate strong binding and the second curve (right of the dotted line) shows a saturable binding plateau. Results suggest specific saturable binding either to different sites on *S. cerevisiae*, or by different mechanisms. **(D)** rSpTrf-E1-FITC binds to specific sites on *V. diazotrophicus*. Binding competition with a fixed saturable concentration of rSpTrf-E1-FITC (as determined in **(B)** and set to 100% fluorescence) and mixed with increasing concentrations of unlabeled rSpTrf-E1 results in decreased fluorescence intensity (FI) of *V. diazotrophicus* by flow cytometry. This indicates that the proteins compete for the same sites. Data are shown as the mean ± 1 SD of three independent experiments. This figure is from Ref. (3). Figure 4E. **(E)** As in **(D)**, competition binding using a saturable level of rSpTrf-E1-FITC [as determined in **(C)** and set to 100% fluorescence] with increasing concentrations of unlabeled rSpTrf-E1 results in decreased FI of *S. cerevisiae* by flow cytometry. Results show two competition curves that correlate with the binding curves in **(C)**. Data are shown as the mean ± 1 SD of three independent experiments. Panels **(B–E)** are reprinted from Ref. (3) with permission from Elsevier.

The association between SpTrf protein expression and immune challenge or injury suggests that these proteins impart important functions in host immune protection. This notion is also based, in part, on the unexpected level of diversity among the *SpTrf* genes, messages, and deduced protein sequences. Although bioinformatic analyses do not detect conserved domains and thus do not provide insights as to possible functions of the proteins, the hypothesis of immune activity has been tested initially with native SpTrf proteins isolated by nickel affinity. SpTrf proteins bind to Gram-negative and Gram-positive bacteria but show variations in binding capabilities among sea urchins (3, 33). Because individual sea urchins can express hundreds of SpTrf protein variants (33, 36), functional characterization of separated SpTrf proteins requires isolated variants. Efforts to achieve expression of six different recombinant SpTrf proteins in a bacterial expression system was successful for only one, suggesting that most of the SpTrf variants are highly toxic and may have antimicrobial activity (3). The single recombinant, rSpTrf-E1 (formerly rSp0032), has an E1 element pattern that is rarely identified among the reported cDNA sequences (2.5% of 688 cDNA sequences) (Figure 9A) (6, 11) and is the first SpTrf protein to be evaluated for function. When rSpTrf-E1 is incubated with two Gram-positive *Bacillus* species, the marine Gram-negative *Vibrio diazotrophicus*, and Baker’s yeast, *Saccharomyces cerevisiae*, saturable binding is observed for *Vibrio* and *Saccharomyces*, but no binding is detected for either of the *Bacillus* species (Figures 9B,C) (3). Competition binding between labeled and unlabeled rSpTrf-E1 indicates specific binding sites on *Vibrio* and *Saccharomyces* (Figures 9D,E), and the two binding curves observed for *Saccharomyces* are also observed for competition binding (Figures 9C,E). These results demonstrate an unexpected outcome of a single protein binding selectively to multiple foreign targets with strong affinity. Furthermore, based on the variations in sequences among the native SpTrf proteins, binding results for rSpTrf-E1 infers that other versions may have different and perhaps overlapping ranges of targets.

**Figure 10:**
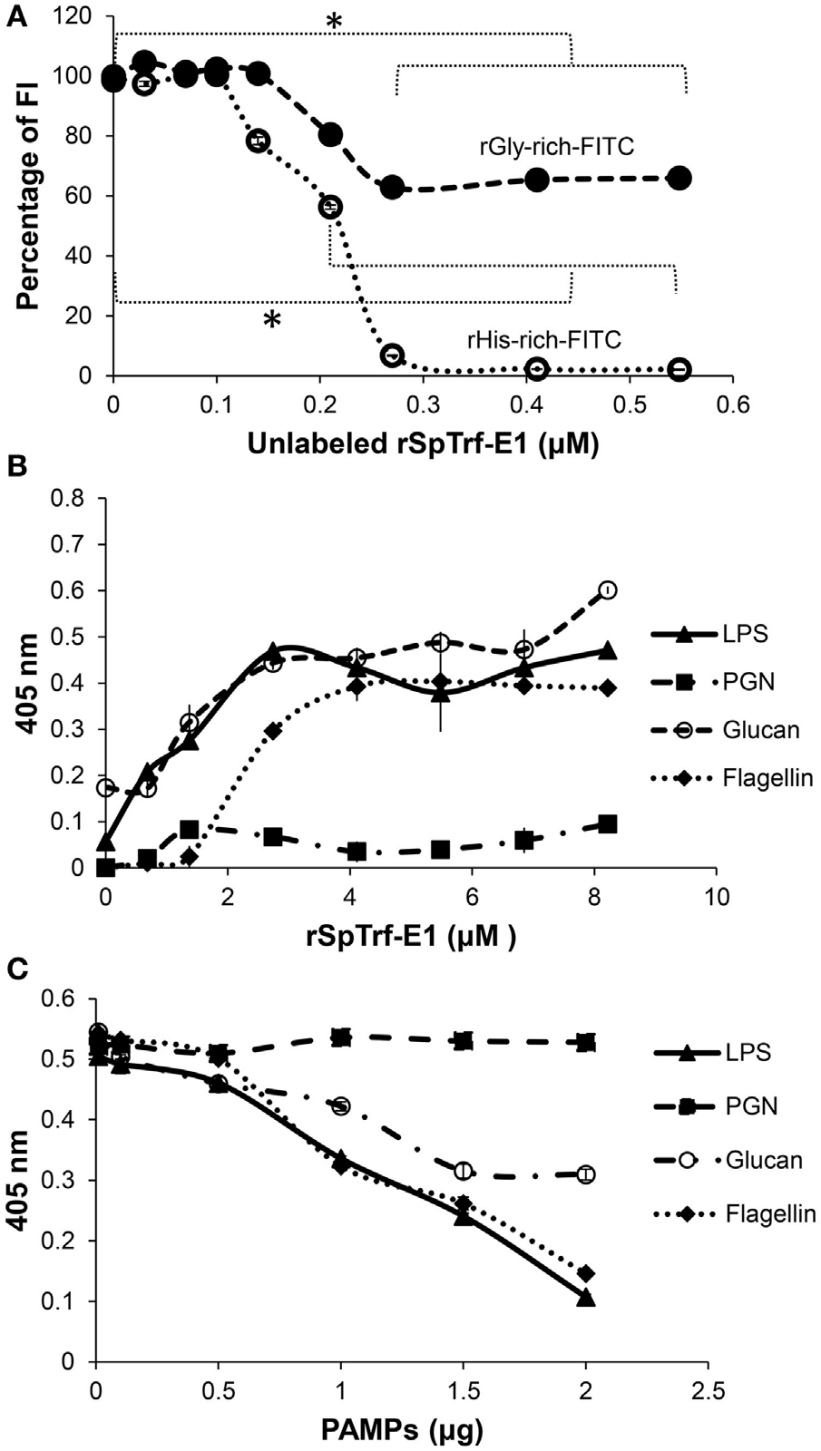
rSpTransformer-E1 (rSpTrf-E1) and the recombinant histidine-rich (rHis-rich) fragment bind to the same sites on yeast, the recombinant glycine-rich fragment (rGly-rich) fragment has expanded binding, and rSpTrf-E1 binds strongly and specifically to several pathogen-associated molecular patterns (PAMPs). **(A)** The full-length rSpTrf-E1 competes for binding sites on *Saccharomyces cerevisiae* with fixed concentrations of both rGly-rich and rHis-rich fragments. Increasing concentrations of rSpTrf-E1 decreases binding by rGly-rich-FITC to yeast by 40% and decreases binding by the rHis-rich-FITC to yeast by 100% as indicated by fluorescence intensity (FI). rSpTrf-E1 and the rHis-rich fragment likely bind to the same sites, whereas the rGly-rich fragment targets additional sites on yeast. **(B)** rSpTrf-E1 binds moderately strongly to lipopolysaccharide (LPS), β-1,3-glucan (glucan), and flagellin but does not bind to peptidoglycan (PGN) as evaluated by ELISA with immobilized PAMPs in wells of a 96-well plate and increasing concentrations of rSpTrf-E1. Binding is detected with an anti-SpTrf (formerly anti-Sp185/333) antibodies followed by Goat-anti-Rabbit-Ig-HRP and measured at 405 nm. Results are shown as the mean ± 1 SD of three independent experiments. **(C)** Preincubation of rSpTrf-E1 with increasing concentrations of various PAMPs in solution interferes with rSpTrf-E1 binding to immobilized LPS. Preincubation with LPS reduces binding to immobilized LPS as expected, and both β-1,3-glucan and flagellin also reduce rSpTrf-E1 binding to immobilized LPS. However, PGN does not interfere. Detection of rSpTrf-E1 bound to LPS in wells is done by ELISA with anti-SpTrf antibodies, Goat-anti-Rabbit-Ig-HRP and measured at 405 nm. Results are presented as the mean ± 1 SD of three independent experiments. These figures are reprinted from Ref. (3) with permission from Elsevier.

SpTransformer proteins share a standard structure (Figure 6A) despite the sequence diversity; however, the differences in the amino acid compositions for the glycine-rich and histidine-rich regions of individual proteins have led to the notion that these regions may have different functions. Consequently, the recombinant fragments of rSpTrf-E1, the recombinant glycine-rich fragment (rGly-rich), recombinant C-terminal end of the gly-rich region (rC-Gly), and recombinant histidine-rich (rHis-rich) fragments (Figure 9A) show different binding characteristics compared to the full-length rSpTrf-E1 when tested against microbial targets (3). The three recombinant fragments bind to all tested foreign cells including the *Bacillus* species indicating altered and broadened binding relative to rSpTrf-E1. The central region of rSpTrf-E1, rC-Gly, multimerizes either in the presence or absence of binding targets and in the absence of other sea urchin proteins. Neither the rGly-rich nor the rHis-rich fragments include the rC-Gly region, and they do not multimerize indicating that this central region of the protein is responsible for multimerization of rSpTrf-E1 and likely for the native SpTrf proteins. The rGly-rich and rHis-rich fragments show similar binding toward *Vibrio* and *Saccharomyces* compared to full-length rSpTrf-E1; however, they both show broadened binding toward the two *Bacillus* species unlike the full-length protein. Binding competition for *Saccharomyces* between the rGly-rich and rHis-rich fragments shows that each reduces binding by the other by 40% suggesting distinct but overlapping binding sites for each fragment. Similarly, when the competitor is the full-length rSpTrf-E1, it reduces binding to *Saccharomyces* by the rGly-rich fragment by 40% and fully competes with the rHis-rich fragment (Figure 10A). These results illustrate that rSpTrf-E1 and the rHis-rich fragment bind to the same sites on yeast, likely with the same mechanisms. However, the rGly-rich fragment when expressed separately binds to additional sites that are not recognized by either rSpTrf-E1 or the rHis-rich fragment. Given mRNA editing and the presence of Gly-rich truncated proteins in the CF [(32) and see Figures 2A and 4B], the broadened binding characteristic suggests possible immune surveillance activities in sea urchins (3). It is apparent that the regions of the full-length SpTrf proteins likely interact and may function together to define binding selectivity to certain target cells.

## rSpTrf-E1 is Intrinsically Disordered and Undergoes Structural Transformation

The multitasking activities of rSpTrf-E1 (i.e., binding to a range of foreign cells) are unique because most other anti-pathogen proteins bind to a single category of foreign cell types and suggest that several molecular targets may be the basis for cellular binding. When rSpTrf-E1 is incubated with *Vibrio*, analysis by gel electrophoresis and mass spectrometry shows that flagellin is co-localized in an SpTrf-positive band. This raises the possibility that binding by rSpTrf-E1 to foreign cells may be mediated through PAMPs (3). In addition to flagellin from *Vibrio*, rSpTrf-E1 also shows strong and specific binding to flagellin from *Salmonella typhimurium*, LPS from *Escherichia coli*, and β-1,3-glucan from *Saccharomyces*, but does not bind to peptidoglycan from *Bacillus subtilis* (Figure 10B). Competition assays among PAMPs shows that binding by rSpTrf-E1 to LPS can be competed by LPS, flagellin, and β-1,3-glucan, but not by peptidoglycan (Figure 10C). This demonstrates that rSpTrf-E1 binds specifically, tightly, and irreversibly to very different types of PAMPs; glucose polymers in β-1,3-glucan, a complex of sugars or lipids in LPS, and amino acids in the non-glycosylated flagellin from *Salmonella*. In contemplating the broad multitasking binding characteristics of rSpTrf-E1, the bioinformatic prediction is that this protein is likely an intrinsically disordered protein (IDP), which is composed of unfolded loops without any ordered relationships and with no secondary structure. This led to the hypotheses that the lack of secondary structure and the possibility of conformational plasticity, or the ability to acquire different sets of secondary folds such as α helices or β strands without energy input, may be a basis for how rSpTrf-E1 may bind and/or interact with such different targets (3, 4). The structural analysis of rSpTrf-E1 by circular dichroism (CD) confirms intrinsic disorder and shows that the protein transforms from disorder to mostly α helical structure in the presence of sodium dodecyl sulfate (SDS), an anionic detergent that is used to simulate anionic environment (49), and 2,2,2-trifluorethanol (TFE), which tends to promote secondary structure of α helices and β strands, and are commonly used reagents in CD studies (Table 2). Furthermore, rSpTrf-E1 readily transforms from disordered to α helical in the presence of LPS. The rGly-rich and rHis-rich fragments also show structural flexibility, but tend to be partially α helical in phosphate buffer, which is not predicted from sequence (4). In the presence of SDS, both the rHis-rich and rGly-rich fragments increase their α helical structure and in TFE both transform to β strand; however, in the presence of LPS, the rGly-rich fragment transforms to β strand and the rHis-rich fragment increases its α helical content (Table 2). These results not only led to the name change from Sp185/333 to SpTransformer to reflect the structural properties of the proteins, but also led to hypotheses for rSpTrf-E1-binding mechanisms. rSpTrf-E1 may have a transient initial binding state that can be established with multiple binding targets and is based on its unique amino acid sequence that is rich in polar and charged amino acids. This characteristic may be responsible for initiating “polyelectrostatic” interactions (50, 51) with negatively charged binding targets on pathogens, perhaps chemically similar to the sulfate group on SDS. The initial interaction may be followed quickly by a secondary step that is based on the hydrophilic nature and structural flexibility of rSpTrf-E1 as an IDP and its transformation to secondary folds for establishing tight binding with multiple targets. Although, the actual underlying chemical mechanism(s) for the binding process remain speculative, the extent of the transformation from disorder to secondary structure may be induced and/or guided by the characteristics of the target. This provides an interesting parallel to an aspect of Linus Pauling’s template theory of antibody formation and the generation of diversity in which direct interactions with an antigen induce the formation of the binding pocket from the unfolded variable domain (52). Since the time of Pauling’s speculations, the mechanisms have been well characterized for generating and selecting for antigen receptors in jawed vertebrates with specific binding only to non-self. Non-rearranging anti-pathogen molecules in both vertebrates and invertebrates also target non-self, but through a wide range of mechanisms. In general, germ-line encoded molecules are evolutionarily selected for binding to PAMPs and not to self. The complexities presented by the SpTrf proteins, including their predicted sequence diversity (6, 11), disordered structure (4, 5), and predictions of *SpTrf* mRNA editing that can change the amino acid sequence or truncate the proteins (30), challenge the concepts of selection for non-self binding by germ-line encoded proteins. Furthermore, these attributes of the *SpTrf* system suggests that the mRNA editing may not be random (see Figure 4A).

**Table 2:** rSpTransformer-E1 (rSpTrf-E1) and the recombinant fragments show changes in secondary structure in different reagents and binding targets.^a^

Protein structure	Secondary structure in different reagents				
	PO_4_	SDS	TFE	LPS	PA
rSpTrf-E1	Disordered	α Helical	α Helical	α Helical	α Helical
% α Helical	1–2^c^	79	95.1	78.5	71.8
Helix tightness^b^		0.59	1	0.66	0.7

Recombinant glycine-rich fragment	α Helical	α Helical	β Strand	β Strand	n/d
% α Helical	15–17	75.1	N/A	N/A	
Helix tightness		0.78	N/A	N/A	

Recombinant histidine-rich fragment	α Helical	α Helical	β Strand	α Helical	n/d
% α Helical	19–30	70.7	46.2	72.8	
Helix tightness		0.76	N/A	0.78	

*^a^These data are from Ref. (4, 5)*.

*PO_4_,10 mM phosphate buffer pH = 7.4; SDS, sodium dodecyl sulfate; TFE, 2,2,2-trifluoroethanol; LPS, lipopolysaccharide from *Escherichia coli*; PA, phosphatidic acid in the form of small vesicles; n/d, not done; N/A, not applicable; the deconvolution to calculate the β strand percentage is not feasible for these samples (4)*.

*^b^Helix tightness is estimated from the *R* value obtained from circular dichroism (CD) spectra and is used to infer the width of an α helical twist. A standard helix has an *R* value of 1. A 3_10_ helix has an *R* value of 0.4, which has a smaller diameter and is longer for a similar number of amino acids (4, 53)*.

*^c^The percentage of secondary structure for either α helix or β strand is deconvoluted from the CD spectra using DichroWeb online server (http://dichroweb.cryst.bbk.ac.uk/html/home.shtml) (54, 55)*.

## rSpTrf-E1 Binds Phosphatidic Acid (PA) and Deforms Membranes

**Figure 11:**
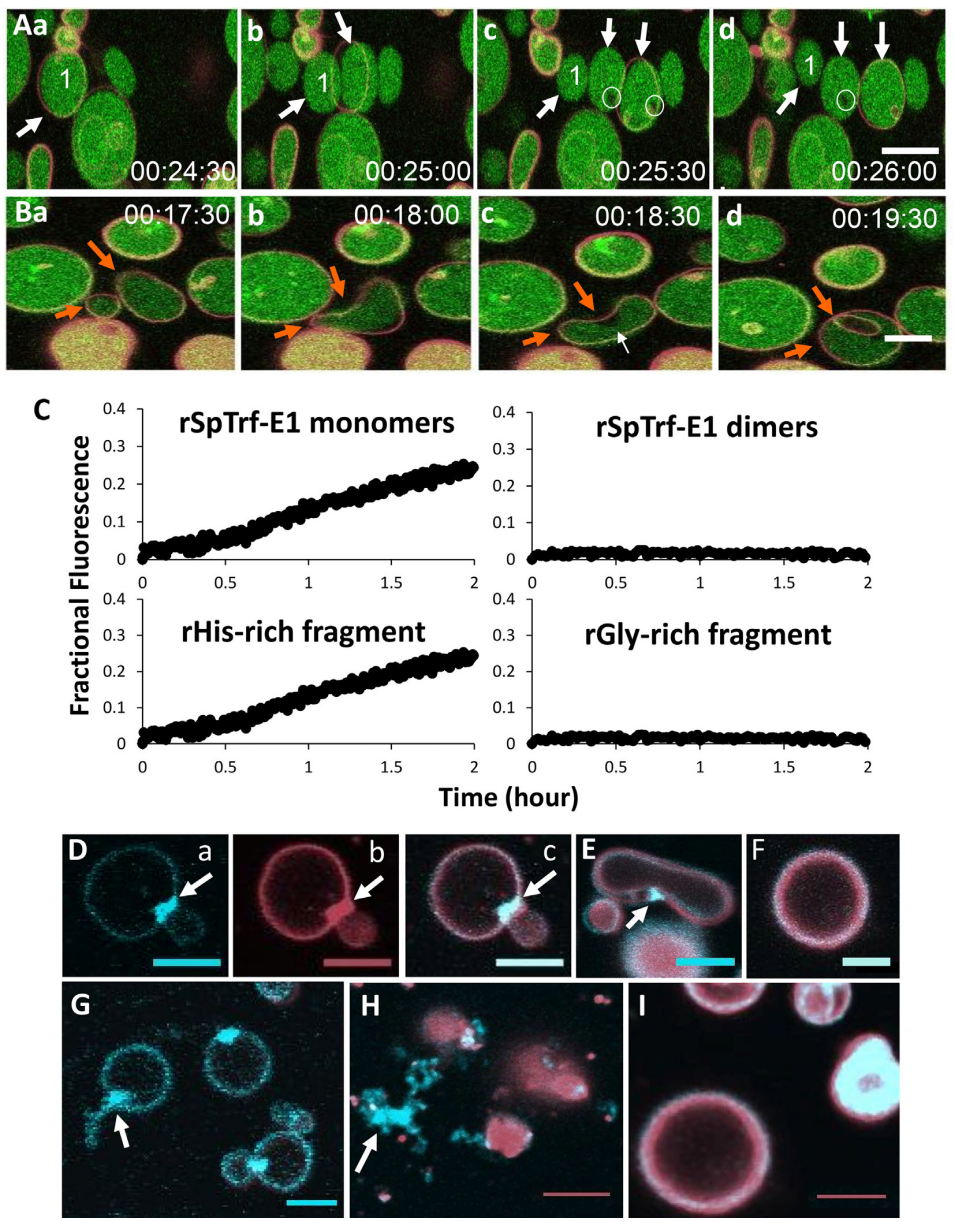
rSpTransformer-E1 (rSpTrf-E1) causes membrane instability and induces liposomes to bud, fuse, invaginate, and leak contents. **(A)** Liposomes composed of 10% phosphatidic acid (PA) and 90% phosphatidylcholine are shown filled with dextran labeled with Alexa Fluor^®^ 488 (green) and the membranes labeled with DiD (red). When in the presence of rSpTrf-E1, liposome labeled #1 shows budding or fission resulting in three liposomes (a–d, arrows). Images were captured by confocal microscopy every 30 s as indicated. Leakage of luminal green dextran is suggested from the black areas in the lumens of some liposomes (c,d, circles). **(B)** Two liposomes of different sizes fuse in the presence of rSpTrf-E1 (a,b, orange arrows). The fused liposome proceeds to invagination (c,d, orange arrows). Note the dark region in the lumen near the convex region of the liposome in (c), which is the site of invagination (d) that forms an internal liposome without luminal dextran labeled with Alexa Fluor^®^ 488 (dextran-488). Images were captured by confocal microscopy every 30 s as indicated. **(C)** Only the monomeric rSpTrf-E1 and the recombinant histidine-rich (rHis-rich) fragment induce dextran-488 leakage from liposomes. Liposomes loaded with 10 mM 8-aminonaphthalene-1,3,6-trisulfonic acid disodium salt (ANTS; fluorescent dye) and 15 mM p-xylene-Bis-pyridinium bromide (DPX; quencher) are incubated with 10 µM recombinant proteins. Luminal leakage separates ANTS from DPX by dilution into the buffer, which is excited at 360 nm and detected at 520 nm as fractional fluorescence relative to the control (lysed to measure 100% release). In the presence of monomeric rSpTrf-E1 and the rHis-rich fragment, luminal content leakage increases over time. Neither dimeric rSpTrf-E1 nor the rGly-rich fragment induce luminal content leakage from liposomes. **(D)** rSpTrf-E1 clusters PA in liposome membranes. A liposome composed of 10% fluorescent blue PA (1-oleoyl-2-{6-[(7-nitro-2-1,3-benzoxadiazol-4-yl)amino]hexanoyl}-sn-glycero-3-phosphate; NBD-PA; a, blue channel) plus the lipophilic dye DiD (b, red channel; c, merge) shows a PA cluster (arrows) at the intersection of two liposomes after 20 min of incubation with rSpTrf-E1. **(E)** NBD-PA is clustered (arrow) at the convex curve in a liposome membrane after 20 min of incubation with rSpTrf-E1. This image is a merge of the blue and red channels. **(F)** A control liposome shows no change in the distribution of NBD-PA after 20 min without rSpTrf-E1. This image is a merge of the blue and red channels. **(G)** Liposomes show clusters of NBD-PA after 20 min in the presence of rSpTrf-E1. One liposome shows extraction of NBD-PA from the membrane (arrow; blue channel only). **(H)** NBD-PA is extracted from liposome membranes after 2 h of incubation with rSpTrf-E1 and forms disordered clusters that are separated from liposomes (arrow). **(I)** Control liposomes show an even distribution of NBD-PA in the liposome membrane after 2 h in the absence of rSpTrf-E1 (merge of blue and red channels). Images in **(A,B,D–I)** were captured by confocal microscopy and all scale bars indicate 10 µm. These figures are reprinted from Ref. (5).

The association of SpTrf proteins with coelomocyte membranes has been well documented (43, 44) but remains a mystery because there are no predicted transmembrane regions or conserved glycophosphatidylinositol linkages from the primary amino acid sequences (11). Consequently, when tested for lipid binding, rSpTrf-E1, the rGly-rich, and the rHis-rich fragments all bind to PA, the rHis-rich fragment also binds weakly to phosphatidylinositol 4 phosphate, and rC-Gly binds weakly to phosphatidylserine (5). PA has a similar amphipathic structure as SDS except it has a phosphate head group, which is the likely binding site as none of the proteins bind to diacylglycerol. rSpTrf-E1 displays the same structural transformation from disordered to α helical in the presence of PA as it does with SDS (Table 2). When PA is incorporated into liposome membranes, rSpTrf-E1 alters liposome morphology, inducing budding or fission, fusion, and invagination (Figures 11A,B). Budding is illustrated by a liposome that buds and forms a total of three liposomes (Figure 11Aa–d; white arrows), fusion is shown between two different sized liposomes that form a single bean-shaped liposome (Figure 11Ba,b; orange arrows), and invagination is illustrated by the bean-shaped liposome that proceeds to a multi-lamellar liposome in which the internal liposome contains no luminal dextran labeled with Alexa Fluor^®^ 488 (dextran-488) (Figure 11Bc,d). The uneven distribution of the luminal dextran-488 noted as dark regions within some liposomes suggests dextran-488 leakage (Figure 11Ac,d; white circles). To verify luminal leakage, liposomes loaded with both ANTS (fluorescent dye) and DPX (quencher) show that rSpTrf-E1 induces fluorescent dye leakage (Figure 11C). Only monomeric rSpTrf-E1 and the rHis-rich fragment induce leakage indicating that the histidine-rich region of the full-length protein is solely responsible for the leakage activity on membranes with PA. It is also noteworthy that pre-dimerized rSpTrf-E1 has no effect on liposomes, suggesting that dimerization and multimerization of the SpTrf proteins deactivate or block their binding activity.

The morphological changes in the liposomes in the presence of rSpTrf-E1 are consistent with the unique structure of PA and the structural change in rSpTrf-E1 from disordered to α helical in the presence of PA (Table 2). PA is a conical phospholipid with a small phosphate head group (56) and its enrichment or clustering in a membrane is known to promote curvature (57). It is noteworthy that the dark luminal region near the convex portion of the liposome membrane in Figure 11Bc (white arrow) suggests leakage and that this is the site of invagination observed 1 min later (Figure 11Bd). These complex morphological changes occur at the same area of the liposome membrane and may be the result of PA bound to rSpTrf-E1. When liposomes composed of blue fluorescently labeled PA (NBD-PA, see legend to Figure 11) and phosphatidylcholine (PC) are incubated with rSpTrf-E1 for 20 min, NBD-PA appears as clusters of bright blue fluorescent patches in the membranes. There is usually a single NBD-PA cluster per liposome, and many are observed at intersections of two liposomes (Figure 11Da–c) and at regions of membranes showing concave curvature (Figure 11E). In one case, an NBD-PA cluster appears in a liposome with an extension from the cluster to outside of the membrane (Figure 11G; arrow). Control liposomes in the absence of rSpTrf-E1 show an even distribution of NBD-PA after 20 min (Figure 11F). When liposomes with NBD-PA are incubated with rSpTrf-E1 for 2 h, NBD-PA appears as disordered tangles outside of the liposome membranes (Figure 11H; arrow), whereas liposomes in the absence of rSpTrf-E1 continue to show an even distribution of NBD-PA in the membranes (Figure 11I). It is likely that the phosphate head group of PA is the binding target for rSpTrf-E1 based on the overall structural similarity to SDS and the amino acid composition of rSpTrf-E1 of which ~25% are positively charged and some or all may be involved with PA binding, although the exact mechanism is not known (5). The hypothesis of structural conformation and plasticity of rSpTrf-E1 is strengthened by the secondary structural changes from disorder to α helical in the presence of PA and the correlated morphological changes in liposomes containing PA. Although these results suggest how one version of the SpTrf proteins may associate with cell membranes, it is unknown whether PA is important for the observed association of SpTrf proteins on the surface of small phagocytes (see Figure 6B) (43). PA is usually present in small quantities in cells but is responsible for many physiological functions as a precursor for synthesis of other phospholipids, part of signaling pathways in response to stress, and other cellular activities (58–61). Although PA is known to be elevated on the cytoplasmic side of the cell membrane for vertebrate phagocytes (62) during phagocytosis (63), it is possible that SpTrf proteins bound to PA on a phagocyte surface may drive membrane curvature for phagocytosis or endocytosis during pathogen clearance (5).

## Conclusion and Overview of the SpTrf System in Sea Urchins

**Figure 12:**
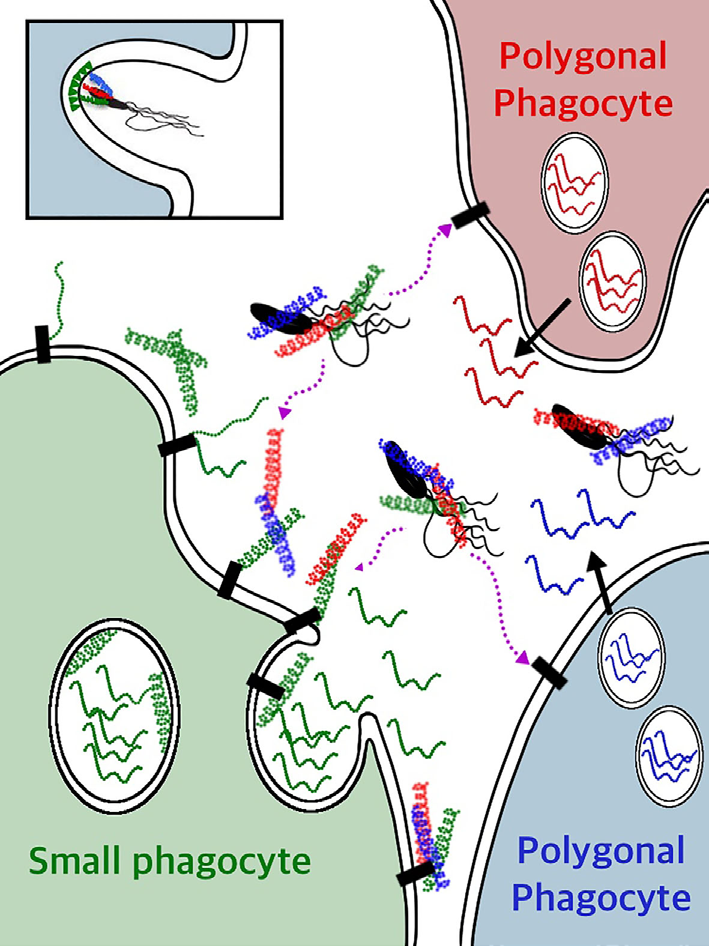
A model for SpTransformer (SpTrf) protein functions for clearance of bacteria from the coelomic fluid (CF). Individual phagocytes secrete a single SpTrf protein variant (41), which is illustrated by individual phagocytes (red, green, blue) producing different (color coded) SpTrf protein variants. Bioinformatic predictions of many deduced SpTrf sequences and circular dichroism results for rSpTransformer-E1 (4) indicate that these proteins are likely intrinsically disordered proteins (IDPs) (squiggles). Upon interaction with or binding to targets in the CF, they transform to α helical structures (corkscrews). Whether the SpTrf proteins associate directly with phospholipids on the surface of small phagocytes (green cell) or whether SpTrf proteins associate with any phagocyte type through putative membrane receptor(s) (black rectangles) remain unknown and await investigation. When vesicle membranes fuse with the cell membrane, the membrane-bound SpTrf proteins are exposed on the surface of the small phagocyte (green cell) (44). Other SpTrf proteins that are secreted by nearby polygonal phagocytes and released into the CF likely bind quickly to pathogens through pathogen-associated molecular patterns (lipopolysaccharide, flagellin, or both) on the pathogen surface and swiftly transform from IDPs to proteins with ordered structure forming helices. Alternatively, secreted SpTrf proteins may bind to the surface of small phagocytes through multimerization with other membrane-bound SpTrf proteins, or may bind directly to phospholipids or to putative receptor(s) (black rectangles). The secreted SpTrf proteins that bind to pathogens may function as opsonins and trigger phagocytosis and pathogen clearance. The insert at the top left illustrates a theoretical clustering of phosphatidic acid (green triangles) in the outer leaflet of a phagocyte plasma membrane (represented as the double black line) by SpTrf proteins bound to the bacterium and induce the concave curvature in the membrane that may aid in the formation of the phagosome and uptake of a microbe. Other mechanisms that are known to be involved with phagosome formation are not shown.

The activities of rSpTrf-E1 and its recombinant fragments show unexpected multitasking activities with tight binding [e.g., *K*_d_ = 0.2 nM for *Vibrio*; (3)] toward certain microbes, PAMPs, and lipids. The recombinant proteins provide new insights into how some of the SpTrf proteins may associate with potential pathogens and, perhaps, with membranes of both sea urchin phagocytes and bacterial surfaces. Activities of rSpTrf-E1 suggest that the sequence diversity of the SpTrf proteins may predict varying ranges of multitasking activities, with possible differing but overlapping activities toward varying groups or species of marine pathogens. We propose an overall model for SpTrf protein function in response to bacterial challenge that attempts to include the results described in this review (Figure 12). Individual phagocytes appear to express a single *SpTrf* gene and produce a single SpTrf protein (41), given minor changes from mRNA editing (30). SpTrf proteins are stored in perinuclear vesicles of phagocytes (Figures 7B–D) (41, 43) and are speculated to be inactive with regard to binding and multimerization. Upon pathogen detection, different SpTrf protein isoforms are secreted into the CF by exocytosis from different phagocytes and may subsequently bind to the surface membrane of small phagocytes (Figure 12; green cell). In addition, the perinuclear vesicles may also contain membrane-bound SpTrf proteins that become associated with the cell surface upon incorporation of the vesicle membrane with the plasma membrane during exocytosis (44). The membrane association of SpTrf proteins may involve a putative membrane receptor(s) rather than or in addition to binding through PA. The SpTrf proteins that are likely secreted as IDPs, bind quickly to pathogens through strong affinity to PAMPs, followed by structural transformation to α helices (Figure 12) or other secondary folds. It is noteworthy that the concentration of SpTrf proteins in the cell-free CF is very low and that nickel-isolated native SpTrf proteins often appear as multimers (33, 36, 43), suggesting that the active proteins have a short half-life as IDPs and either bind to pathogens or multimerize and are inactivated (3) (see Figure 11C). We hypothesize that multimerization of different SpTrf variants secreted from different phagocytes occurs upon pathogen binding and opsonization that leads to pathogen clearance by triggering phagocytosis through putative receptor(s) (potentially including PA) on the polygonal phagocytes (Figure 12). In support of this hypothesis, HeTrf proteins have been observed in phagosomes in association with bacteria in the sea urchin, *H. erythrogramma* (44). Alternatively, there may be membrane-bound SpTrf proteins on phagocytes that function as putative receptors for SpTrf proteins that have opsonized bacteria. The subsequent multimerization among proteins on both the microbe and the coelomocyte surface may lead to phagocytosis. This notion is particularly interesting if PA is present on the coelomocyte plasma membrane and is clustered as a result of SpTrf binding to induce membrane curvature, which would assist with progression to phagocytosis (Figure 12; top left insert).

## The SpTrf System has Multiple Levels of Diversification

The host–pathogen arms race drives diversification of pathogens to improve their abilities to infect, proliferate, disseminate, and survive. The requirement for the host to survive the arms race also drives diversification mechanisms of the host immune system to detect and respond to constantly changing pathogens (1, 16, 64). The best example of host immune diversification is the well-understood vertebrate somatic recombination of the Ig and TcR genes that function in immune detection and response and that are diversified by the recombinase enzymes encoded by the *RAG1/2* genes (65, 66). Interest in the evolutionary origins of the *RAG*s has led to the identification of homologs in a few invertebrates (67–69). *SpRAG1L* and *SpRAG2L* homologs are present and linked in the sea urchin genome, are expressed in embryos and coelomocytes (67), and the SpRAG1L enzyme functions with mouse RAG2 to generate a low level of DNA recombination (70). Although intriguing, it is not clear whether SpRAG1L and SpRAG2L function together in sea urchin cells, and neither the DNA sequences that they may recognize nor the genes that they may impact are known. Although swift changes in the *SpTrf* gene family structure and diversity may be considered as theoretical connections to SpRAGL recombinase activity, it is not known whether these enzymes are involved in changes in the diversity of this gene family.

**Figure 13:**
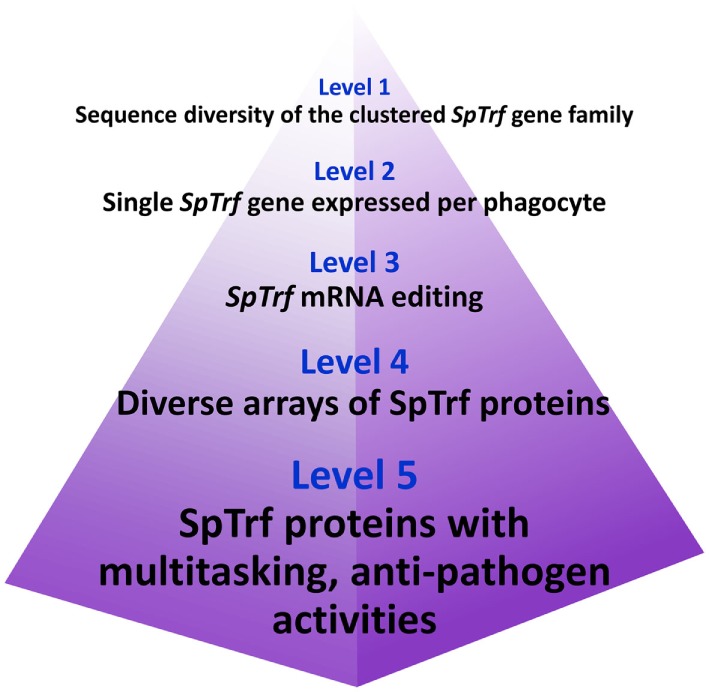
Five levels of diversification in the SpTransformer (SpTrf) system.

The diversity of the SpTrf system has been attributed to five levels of diversification with the beneficial outcome of generating a range of SpTrf proteins in the CF that extend beyond the diversity of the *SpTrf* gene family encoded in the genome (Figure 13). Level 1: the sequence diversity among the members of the *SpTrf* gene family, including the structure of the family in clusters of genes with shared sequences, in addition to possible gene conversion, segmental duplications, and putative gene deletions that appear to be associated with STRs, suggest localized genomic instability that may be required for gene diversification in this system (2, 28, 29). Genomic instability is consistent with differences in the members of the *SpTrf* gene family among sea urchins (29). Level 2: *SpTrf* gene expression from single phagocytes has inferred that only a single *SpTrf* gene is expressed per cell (41). This leads to the hypothesis that variations in the *cis* and/or *trans* regulatory regions associated with the *SpTrf* genes may control whether specific or subsets of genes are expressed (or repressed) in phagocyte responses to particular pathogens or categories of pathogens. This putative second level of gene expression control could limit or target the diversity of the expressed proteins to optimize protection against particular pathogens and is expected to require coordination among responding and non-responding phagocytes. Level 3: the prediction of mRNA editing increases the diversity of the mRNAs particularly when they are translated (edited or not) to both full-length and truncated proteins that may include missense sequence (30, 32). Editing is expected to expand the diversity of the proteins relative to the sequences encoded by the genes, including the possibility of expanded binding capabilities for truncated SpTrf proteins that are missing the histidine-rich region (3, 11). The increased presence of edited mRNAs encoding truncated and/or missense proteins prior to immune challenge suggests an active, non-random editing process with an outcome of altered functions for truncated proteins. Level 4: the diverse arrays of SpTrf proteins are the outcome of the diversification processes described in the preceding levels, which are putatively broadened further by posttranslational modifications that may alter protein function. These types of modifications have been suggested from the arrays of SpTrf proteins with the same molecular weight but with wide ranges of pI and *vice versa* (33). This may be the result of a number of types of posttranslational changes to proteins including multimerization, glycosylation for which there are a number of conserved linkage sites within and among the SpTrf isoforms (6), in addition to possibilities for phosphorylation and acetylation (33). Level 5: the new diversification level for this system is the unexpected range of rSpTrf-E1 protein functions and its unusual structural characteristics that may apply to many, if not most of the SpTrf proteins (3–5). The variety of SpTrf proteins that are expressed in response to a particular pathogen may each display differing but also overlapping ranges of multitasking activities that are based on the hydrophilic character of the proteins, the prediction that they are flexible IDPs, and the expectation that they undergo structural transformation upon binding to a range of targets. Nickel-isolated native SpTrf proteins bind to bacteria and yeast (3) and may function as opsonins to augment phagocytosis. The ability to bind selectively and tightly to multiple PAMPs is likely to confound the abilities of potential marine pathogens and opportunists to alter simultaneously multiple molecular attributes to avoid recognition, opsonization, and possible killing by the SpTrf proteins. These multiple levels of diversification plus the flexibility and the predicted multitasking activities of SpTrf proteins are novel solutions in the immunological arms race and provide evidence for how this immune protein family may act as an extraordinarily effective component of the immune system in echinoids.

## Author Contributions

LCS and CML wrote, edited, and approved the manuscript.

## Conflict of Interest Statement

The authors declare that they have no conflicts of interest and that writing this review was conducted in the absence of any commercial or financial relationships that could be construed as a potential conflict of interest.

## Abbreviations

2D, two dimensional; BAC, bacterial artificial chromosome; CD, circular dichroism; CF, coelomic fluid; aCF, artificial CF; dextran-488, dextran labeled with Alexa Fluor^®^ 488; dsRNA, double stranded RNA; EST, expressed sequence tag; gDNA, genomic DNA; HeTrf, the transformer family from *Heliocidaris erythrogramma*; IDP, intrinsically disordered protein; IQ, immunoquiescent; PA, phosphatidic acid; PC, phosphatidylcholine; r, recombinant; rC-Gly, recombinant C-terminal end of the glycine-rich region; RGD, arginine, glycine, aspartic acid motif; rGly-rich, recombinant glycine-rich fragment; rHis-rich, recombinant histidine-rich fragment; rSpTrf-E1, recombinant SpTrf protein with an E1 element pattern; SDS, sodium dodecyl sulfate; *SpRAG1L*, recombinase activating gene 1-like homolog from *Strongylocentrotus purpuratus*; *SpRAG2L*, recombinase activating gene 2-like homolog from *Strongylocentrotus purpuratus*; SpTrf, the transformer family from *Strongylocentrotus purpuratus*; STRs, short tandem repeats; TFE, 2,2,2-trifluorethanol.
